# Glucose induces rapid changes in the secretome of *Saccharomyces cerevisiae*

**DOI:** 10.1186/1477-5956-12-9

**Published:** 2014-02-12

**Authors:** Bennett J Giardina, Bruce A Stanley, Hui-Ling Chiang

**Affiliations:** 1 Department of Pharmaceutical Sciences, School of Pharmacy, University of Maryland, HSF II, 20 Penn Street, Baltimore, Maryland 21201-1140, USA; 2 Section of Research Resources, Penn State University College of Medicine, 500 University Drive, Hershey, PA 17033, USA; 3 Department of Cellular and Molecular Physiology, Penn State University College of Medicine, 500 University Drive, Hershey, PA 17033, USA

## Abstract

**Background:**

Protein secretion is a fundamental process in all living cells. Proteins can either be secreted via the classical or non-classical pathways. In *Saccharomyces cerevisiae*, gluconeogenic enzymes are in the extracellular fraction/periplasm when cells are grown in media containing low glucose. Following a transfer of cells to high glucose media, their levels in the extracellular fraction are reduced rapidly. We hypothesized that changes in the secretome were not restricted to gluconeogenic enzymes. The goal of the current study was to use a proteomic approach to identify extracellular proteins whose levels changed when cells were transferred from low to high glucose media.

**Results:**

We performed two iTRAQ experiments and identified 347 proteins that were present in the extracellular fraction including metabolic enzymes, proteins involved in oxidative stress, protein folding, and proteins with unknown functions. Most of these proteins did not contain typical ER-Golgi signal sequences. Moreover, levels of many of these proteins decreased upon a transfer of cells from media containing low to high glucose media. Using an extraction procedure and Western blotting, we confirmed that the metabolic enzymes (glyceraldehyde-3-phosphate dehydrogenase, 3-phosphoglycerate kinase, glucose-6-phosphate dehydrogenase, pyruvate decarboxylase), proteins involved in oxidative stress (superoxide dismutase and thioredoxin), and heat shock proteins (Ssa1p, Hsc82p, and Hsp104p) were in the extracellular fraction during growth in low glucose and that the levels of these extracellular proteins were reduced when cells were transferred to media containing high glucose. These proteins were associated with membranes in vesicle-enriched fraction. We also showed that small vesicles were present in the extracellular fraction in cells grown in low glucose. Following a transfer from low to high glucose media for 30 minutes, 98% of these vesicles disappeared from the extracellular fraction.

**Conclusions:**

Our data indicate that transferring cells from low to high glucose media induces a rapid decline in levels of a large number of extracellular proteins and the disappearance of small vesicles from the extracellular fraction. Therefore, we conclude that the secretome undergoes dynamic changes during transition from glucose-deficient to glucose-rich media. Most of these extracellular proteins do not contain typical ER signal sequences, suggesting that they are secreted via the non-classical pathway.

## Background

Protein secretion is an important process for both prokaryotic and eukaryotic cells. Secretory proteins include growth factors, inflammatory cytokines, coagulation factors, extracellular matrix proteins, proteases, and protease inhibitors [1-9]. As such, they participate in various physiological processes such as immune defense, blood coagulation, cell growth, cell differentiation, and proliferation [1-5,7-9]. For fungi that have thick cell walls, secretory proteins are involved in the formation and maintenance of cell walls, cell separation, and nutrient scavenging [10-17]. Proteins that are secreted from viruses, bacteria, fungi, and parasites are important for pathogen-host interactions [18-25]. Furthermore, secretory proteins also play crucial roles in cancer angiogenesis, differentiation, invasion, and metastasis [6,7,26-29].

Proteins can be secreted from cells via the classical and non-classical pathways [2,3,8,9,23,30,31]. In the classical pathway, proteins that contain a specific N-terminal signal sequence are translocated into the ER, transported to the Golgi and then secreted by secretory vesicles [30,32,33]. Accumulating evidence indicates that a large number of signal-less proteins can also be secreted through the non-classical pathway [1,2,4,5,10,21,23,31], [34], including metabolic enzymes, chaperones, translation factors, and transcriptional regulators [5,8,9,11,20-24,31,34-37]. Glyceraldehyde-3-phosphate dehydrogenase (GAPDH) is a glycolytic enzyme and has been shown by immuno-transmission electron microscopy (immuno-TEM) to be present on the surface of *Candida albicans* and *Saccharomyces cerevisiae*[14]. Using the immuno-TEM technique, another glycolytic enzyme, enolase, was also found on the surface of *Listeria monocytogenes*[24]. In systemic infection by *Candida albicans*, enolase and GAPDH are secreted [11,14,16,38]. GAPDH on the surface of *Candida albicans* is enzymatically active and binds to the host proteins fibronectin and laminin [14,16,38]. Furthermore, GAPDH and Hsp70 have been recently identified as major antigens in sera from patients infected with *Echinostoma friedi*[19].

Gluconeogenic enzymes have been identified in multiple secretomic studies from bacteria and parasites [18,22-24,37]. Fructose-1,6-bisphosphatase is found in the secretomes from *Bacillus anthracis*[18] and *Clonorchis sinensis*[37], while isocitrate lyase is detected in the secretome in *Bacillus anthracis*[18]. Moreover, malate dehydrogenase is present in the secretomes from *Bacillus anthracis*[18], *Clonorchis sinensis*[37], and *Schistosoma mansoni*[22]. Phosphoenolpyruvate carboxykinase is also detectable in the secretomes from *Clonorchis sinensis*, *Echinostoma caproni,* and *Schistosoma mansoni*[22,23,37].

The yeast *Saccharomyces cerevisiae* is an excellent model system to study global changes in transcription, translation, and protein levels in response to environmental changes such as the availability of nutrients [39-44]. Although yeast can utilize both fermentable and non-fermentable carbon sources, glucose is the preferred carbon source. It has profound effects in gene transcription, translation, mRNA stabilities, and enzymatic activities [45-50]. For example, glucose leads to inactivation of gluconeogenic enzymes, mitochondrial proteins, and enzymes involved in the metabolism of acetate, maltose, glycerol, and galactose [51-55]. Inactivation of gluconeogenic enzymes during glucose re-feeding prevents energy futile cycles that are detrimental to cells. The key gluconeogenic enzyme fructose-1,6-bisphosphatase (Fbp1p) has been used extensively to study glucose-induced degradation [53,56-59]. Fbp1p is either ubiquitinated and degraded in the proteasome [60,61], or phosphorylated and degraded in the vacuole [53,56-59]. Importantly, the site of Fbp1p degradation is dependent on the duration of starvation [62]. For the vacuole pathway, gluconeogenic enzymes including Fbp1p, malate dehydrogenase (Mdh2p), isocitrate lyase (Icl1p), phosphoenolpyruvate carboxykinase (Pck1p), and malate synthase (Mls1p) were in the extracellular fraction during growth in low glucose. Furthermore, their levels in the extracellular fraction were reduced following a transfer of cells to media containing high glucose. This decrease is dependent on the presence of glucose in the media. When cells were transferred to media without glucose, these proteins did not decrease levels in the extracellular fraction. We hypothesized that changes in the secretome induced by glucose were not limited to gluconeogenic enzymes. The goals of this study were to use the iTRAQ approach to test our hypothesis and to identify proteins in the secretome whose levels changed upon a transfer of cells from low to high glucose media.

Here, we report the identification of 347 extracellular proteins from two independent iTRAQ experiments. This included metabolic enzymes and proteins involved in oxidative stress, translation, protein folding, and proteins with unknown functions. Most of these proteins did not contain the N-terminal ER signal sequence. Many of these identified proteins are also commonly found in secretomic studies from bacteria, fungi, parasites, and human cells [19,20,28,35]. Using an extraction procedure and Western blotting, we confirmed that metabolic enzymes (glyceraldehyde-3-phosphate dehydrogenase, 3-phosphoglycerate kinase, glucose-6-phosphate dehydrogenase, pyruvate decarboxylase), proteins involved in oxidative stress (superoxide dismutase and thioredoxin), and heat shock proteins (Ssa1p, Hsc82p, and Hsp104p) were present in the extracellular fraction in cells grown in low glucose. The extracellular level of these proteins was rapidly reduced following a transfer of cells from low to high glucose media. Furthermore, we performed TEM studies and observed numerous small vesicles in total extracts isolated from cells grown in low glucose. Following a shift of cells to media containing high glucose for 30 min, most of these vesicles disappeared. We conclude that the secretome undergoes dynamic changes during transition from glucose-deficient to glucose-rich media.

## Results

### Experimental conditions to study the effects of glucose on protein levels

**Figure 1:**
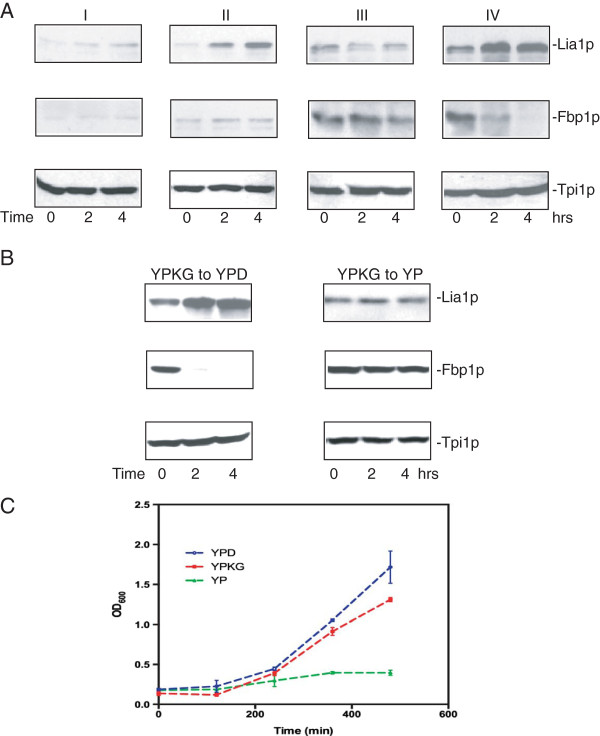
**Experimental conditions to study glucose effects in regulating protein levels. A**, wild-type cells expressing Lia1p-GFP were grown either in YNB-based media containing 2% glucose (I and II) or in YPKG-based media containing 0.5% glucose (III and IV) for 3d. In experiment I, 2% glucose was added directly to the existing YNB culture for 0, 2, and 4 hours. In experiment II, cells were grown in YNB-based media, pelleted and resuspended in fresh YNB with 2% glucose for 0, 2, and 4 hours. In experiment III, cells were grown in YPKG for 3d and 2% glucose was added directly to the existing YPKG media for 0, 2, and 4 hours. In experiment IV, cells were grown in YPKG for 3d, pelleted, and resuspended in YPD media for 0, 2, and 4 hours. Levels of Lia1p-GFP, Fbp1p, and Tpi1p were examined using anti-GFP, anti-Fbp1p, and anti-Tpi1 antibodies. **B**, wild-type cells were grown in YPKG for 3d and transferred to YPD (left panels) or YP (right panels) for 0, 2, and 4 hours. Levels of Lia1p, Fbp1p, and Tpi1p were examined by Western blotting using anti-GFP, Fbp1p, and Tpi1p antibodies. **C**, wild-type cells were grown in YPKG for 3d and diluted to OD_600_ = 0.2/ml in YPD (2% glucose), YPKG (0.5% glucose), or YP (0% glucose). Cell densities were measured at OD_600_ for 0, 2, 4, 6, and 8 hours using a Beckman spectrophotometer.

Glucose has profound effects in regulating proteins levels. For instance, glucose up-regulates Lia1p involved protein synthesis, while down-regulating enzymes involved in gluconeogenesis. There are different ways to examine glucose effects in regulating protein levels. We used wild-type cells grown either in YNB-based media (yeast nitrogen base with amino acids) or YP based media to study glucose effects in up-regulation of Lia1p and down-regulation of Fbp1p (Figure 1A). In Experiment I, wild-type cells expressing Lia1p-GFP were grown in YNB-based media containing 2% glucose for 3d followed by the addition of 2% glucose directly to the existing culture media for 0, 2, and 4 hours. In experiment II, cells were grown in YNB media containing 2% glucose for 3d. Cells were pelleted and resuspended in fresh YNB with 2% glucose for 0, 2, and 4 hours. In experiment III, cells were grown in YP-based media containing 0.5% glucose (YPKG) for 3d followed by the addition of 2% glucose directly to the existing YPKG media for 0, 2, and 4 hours. In experiment IV, cells were grown in YPKG for 3d. Cells were pelleted and resuspended in YPD media containing 2% glucose for 0, 2, and 4 hours. Equal amounts of cells were harvested at each time point, processed and examined for changes in Lia1p and Fbp1p levels (Figure 1A). In experiments I and II where cells grown in YNB-based media, the level of Liap1 was low with an increase at t = 4 hours. Lia1p levels were higher in cells grown in YPKG. However, Lia1p was not up-regulated in experiment III but was up-regulated rapidly in experiment IV. Fbp1p levels were low in cells grown in YNB at t = 0 and did not show significant changes at the 2 and 4 hour time points (experiments I and II). Fbp1p levels were higher in cells grown in YPKG (experiments III and IV). Additionally, Fbp1p levels decreased slowly in experiment III but decreased rapidly in experiment IV. Tpi1p (triose phosphate isomerase) is a glycolytic enzyme and levels of this protein were similar under all four experimental conditions. Because experiment IV produced a faster increase in Lia1p levels and a faster decrease in Fbp1p levels, this condition was used to study glucose effects for most experiments described in this study unless otherwise specified.

To further determine whether or not changes in Lia1p and Fbp1p levels were due to the presence of glucose in the media, wild-type cells expressing Lia1p-GFP were grown in YPKG and transferred to YPD or YP (without added glucose) for 0, 2, and 4 hours (Figure 1B). Again, when cells were transferred from YPKG to YPD, levels of Lia1p increased and levels of Fbp1p decreased (Figure 1B, left panels). However, when cells were transferred from YPKG to YP, Lia1p levels did not increase and Fbp1p levels did not decrease (Figure 1B, right panels). Therefore, the presence of 2% glucose in the YPD media is critical in up-regulating Lia1p and down-regulating Fbp1p.

We next examined whether or not cells grown in YPKG media for 3 days were able to re-grow in YPD media containing 2% glucose (Figure 1C). Wild-type cells were grown in YPKG for 3d to OD_600_ = 4-5/ml. Cells were diluted to OD_600_ = 0.2/ml in YPD containing 2% glucose or YP media without glucose for 0–8 hours and changes in cell densities were measured at OD_600_ using a spectrophotometer. When cells were diluted in YPD, cells increased their densities after a lag. Likewise, when cells were diluted in YPKG, cells densities also increased after a lag. Hence, cells are able to resume growth when diluted in YPD or YPKG. In contrast, there was little increase in density when cells were cultured in YP media without glucose, suggesting that the presence of glucose in the media is required for cells to re-grow.

### Fbp1p is re-distributed from the periplasm/extracellular fraction to the cytoplasm when cells are transferred from YPKG to YPD

**Figure 2:**
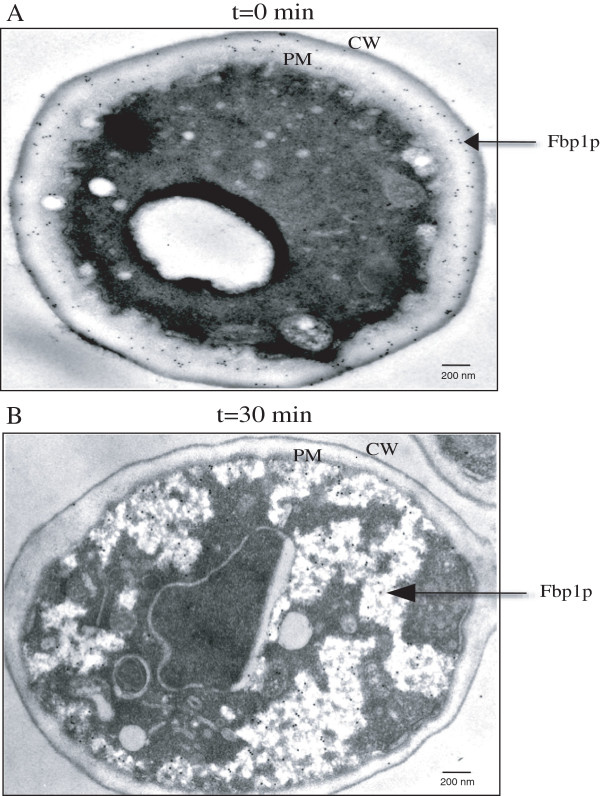
**Fbp1p is re**-**distributed from the periplasm to the cytoplasm upon a transfer of cells from YPKG to YPD.** Wild-type cells were grown in YPKG for 3d (A, t = 0 min) or transferred to YPD for 30 min (B, t = 30 min). Cells were processed and Fbp1p was visualized by immuno-TEM. The number of gold particles in the cytoplasm and the periplasm was 26 and 162 in t = 0 min wild-type cells **(A)**, and 146 and 3 in t = 30 min wild-type cells **(B)**, respectively. Bars: 200 nm, PM: plasma membrane, CW: cell wall.

We next determined the effects of glucose on the distribution of Fbp1p at the ultra-structural level using immuno-TEM. Wild-type cells were grown in YPKG for 3d and transferred to YPD for 30 min. Cells were processed and thin sections of cells were incubated with affinity purified anti-Fbp1p antibodies followed by goat anti-rabbit secondary antibodies conjugated with 10 nm gold particles. The distribution of Fbp1p was then observed by TEM. When cells were grown in YPKG for 3d, substantial amounts of Fbp1p were in the periplasm (Figure 2A, arrow). Because Fbp1p is degraded in the vacuole following glucose addition, extracellular Fbp1 should be internalized upon a transfer or cells from YPKG to YPD. Indeed, Fbp1p was found in intracellular structures that contained clusters of small vesicles at the t = 30 min time point (Figure 2B, arrow). Thus, transferring cells from low to high glucose causes a rapid redistribution of Fbp1p from the periplasm to the cytoplasm, resulting in a rapid decline in Fbp1p levels in the extracellular fraction.

### Extraction of extracellular proteins

We next used an extraction procedure to determine whether or not this protocol could detect a decline in Fbp1p levels in the extracellular fraction*.* This protocol utilizes the combination of reducing agents such as β-mercaptoethanol (βME) or dithiothreitol (DTT) and high pH to release proteins in the extracellular fraction [13,63]. This method of extraction has been utilized to study the secretion of mammalian galectin-1 expressed in *Saccharomyces cerevisiae*[64]. A similar method has been used to identify proteins associated with the cell wall in *Candida albicans*[16,38].

**Figure 3:**
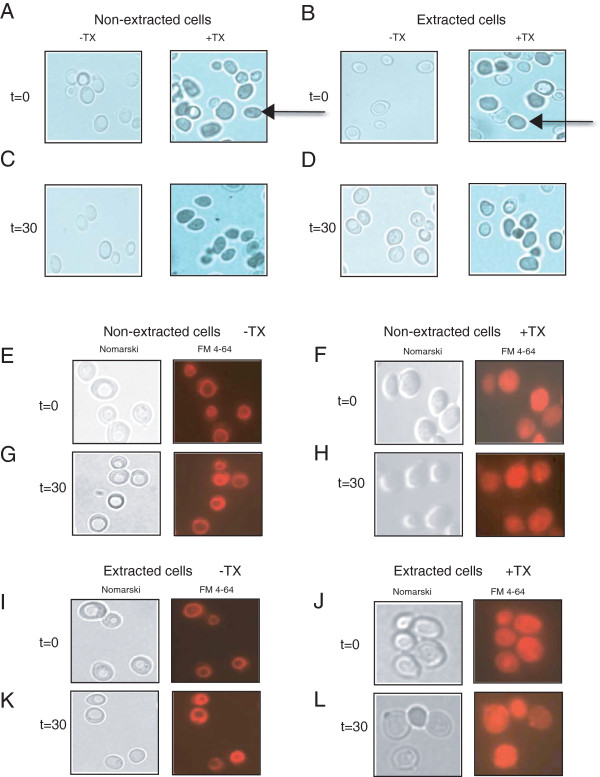
**Extracted cells transport the vital dye FM to the vacuole membrane. (A-D)**, wild-type cells were grown in YPKG for 3d (t = 0) or transferred to YPD for 30 min (t = 30). Cells were divided and half of the cells were extracted. Non-extracted and extracted cells at the t = 0 and t = 30 min time points were incubated with or without 2% Triton X-100 (TX) for 30 min followed by incubation with trypan blue for 30 min. Cells were examined using a light microscope. **(E-L)**, wild-type cells were grown in YPKG for 3d (t = 0) or transferred to glucose for 30 min (t = 30) and harvested. Cells were incubated in the absence or presence of Triton X-100 (TX) and incubated with FM for 3 hours. The distribution of FM was observed by fluorescence microscopy in non-extracted t = 0 cells **(E)**, t = 30 cells **(G)**, TX treated t = 0 cells **(F)**, and TX treated t = 30 cells **(H)**. The same amounts of t = 0 and t = 30 min cells were subjected to the extraction procedure and treated in the absence **(I and K)** or presence of Triton X-100 **(J and L)**. Cells were incubated with FM and the distribution of FM was examined by fluorescence microscopy.

We first determined whether or not cells that were subjected to the extraction procedure retained the ability to exclude trypan blue from cells (Figure 3A-D). Trypan blue dye permeates dead cells and is therefore an indicator of cell viability. Wild-type cells were grown in YPKG for 3d (t = 0) or transferred to YPD for 30 min (t = 30). Cells were then treated with or without Triton X-100 and incubated with trypan blue for 30 min. Cells were then observed by light microscopy. In t = 0 and t = 30 cells, the dye did not stain cells (Figure 3A and C, left panels). However, when Triton X-100 was added to t = 0 and t = 30 cells followed by incubation with trypan blue, about 60-90% of the cells were stained (Figure 3A and 3C, right panels). We also used t = 0 and t = 30 cells that were extracted and then incubated with trypan blue. The majority of extracted cells were not stained (Figure 3B and 3D, left panels). In contrast, when Triton X-100 was added to the extracted cells followed by incubation with trypan blue, about 60-90% of the cells were stained (Figure 3B and 3D, right panels).

We also examined whether or not cells that were extracted retained the ability to internalize exogenous molecules into the cells. FM 4–64 (FM) is a vital dye that is internalized into live cells and actively transported to the vacuole via the endocytic pathway [65]. Wild-type cells were grown in YPKG for 3d (t = 0) or transferred to glucose for 30 min (t = 30). Cells were treated with or without Triton X-100 and then incubated with FM for 3 hours. The same amounts of cells were also subjected to the extraction protocol and treated in the absence or presence of Triton X-100 followed by incubation with FM for 3 hours. The distribution of FM was then observed using fluorescence microscopy (Figure 3E- 3L). In t = 0 and t = 30 non-extracted cells, this dye was transported to the vacuole membrane as ring-like circles (Figure 3E and 3G). In contrast, when these cells were treated with Triton X-100 and then incubated with FM, this dye was not on the vacuole membrane (Figure 3F and 3H). In t = 0 and t = 30 cells that were extracted and then incubated with FM, this dye was also transported correctly to the vacuole membrane (Figure 3I and 3K). However, when extracted cells were treated with Triton X-100 and incubated with FM, this dye was not on the vacuole membrane (Figure 3J and 3L). Thus, FM is targeted correctly to the vacuole membrane in both non-extracted and extracted cells. Furthermore, the endocytic pathway is functional in cells that were either grown in YPKG or transferred to YPD for 30 min.

**Figure 4:**
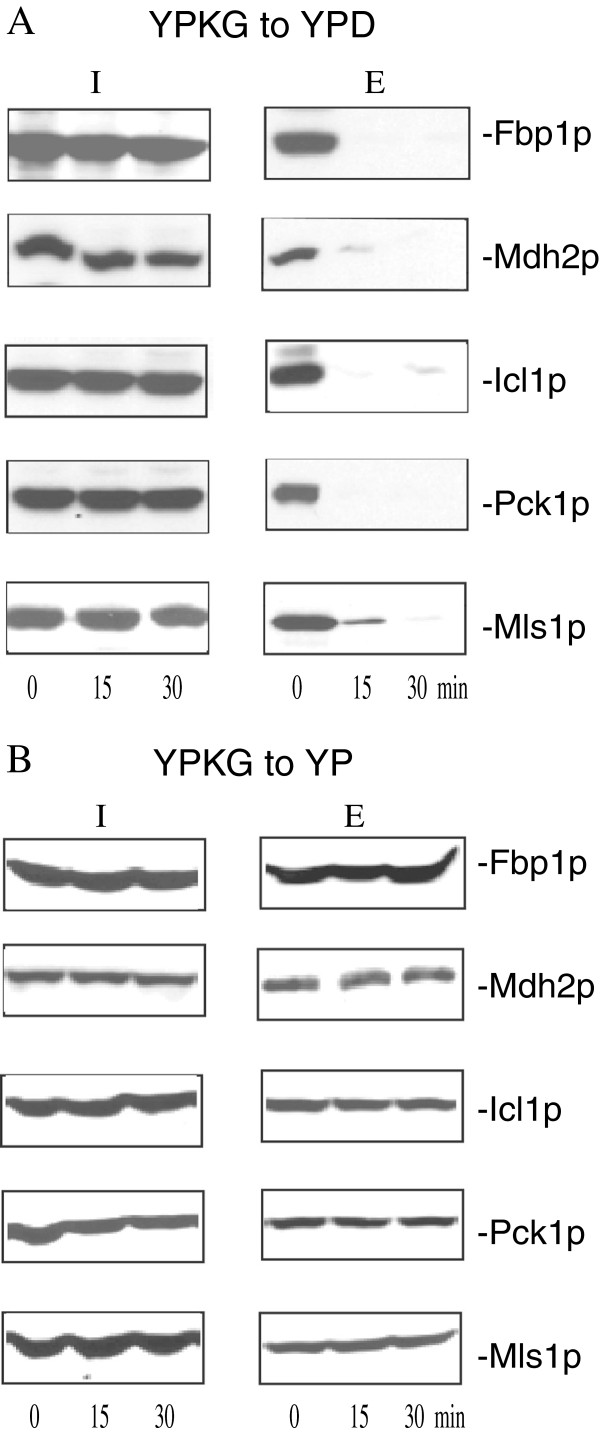
**Glucose is required for the decrease of gluconeogenic enzymes in the extracellular fraction. (A)** Wild-type cells expressing either Fbp1p-GFP, Mdh2p-GFP, Icl1p-GFP, Pck1p-GFP, or Mls1p-GFP were grown in YPKG media for 3d. Cells were harvested or transferred to YPD media for 0, 15, and 30 minutes and harvested. The distribution of GFP tagged proteins in the intracellular (I) and extracellular (E) fractions were determined using Western blotting with anti-GFP antibodies. **(B)**, Wild-type cells expressing either Fbp1p-GFP, Mdh2p-GFP, Icl1p-GFP, Pck1p-GFP, or Mls1p-GFP were transferred from YPKG to YP media containing 0% added glucose for 0, 15, and 30 minutes. Levels of GFP tagged proteins in the intracellular (I) and extracellular (E) fractions were determined using Western blotting with anti-GFP antibodies.

We next used the extraction procedure to examine whether or not this protocol could detect a decline in Fbp1p levels in the extracellular fraction when cells were transferred from YPKG to YPD. Wild-type cells expressing GFP-tagged proteins were grown in YPKG media for 72 hours and aliquoted. The same amounts of cells were then transferred to YPD media containing 2% glucose (Figure 4A) or to YP media containing no glucose (Figure 4B) for 0, 15, and 30 min. Following the extraction procedure, the distribution of GFP-tagged proteins in the intracellular and extracellular fractions was determined by Western blotting with anti-GFP antibodies (Figure 4A). In this study, proteins that were released into the supernatant following the extraction procedure were precipitated with TCA, washed and solubilized in SDS sample buffer to form the extracellular fraction. Proteins from the cell-associated fraction were solubilized in SDS sample buffer and this was called the intracellular (I) fraction in this study. Fbp1p was in the extracellular fraction at t = 0 min. Levels of this protein in the extracellular fraction decreased rapidly by t = 30 min (Figure 4A). Other gluconeogenic enzymes are also degraded in the vacuole in response to glucose re-feeding [62,66]. Therefore, they should show distribution patterns similar to those observed for Fbp1p. These gluconeogenic enzymes include malate dehydrogenase (Mdh2p which catalyzes the interconversion of malate and oxaloacetate), isocitrate lyase (Icl1p which produces succinate and glyoxylate from isocitrate), phosphoenolpyruvate carboxykinase (Pck1p which coverts oxaloacetate to phosphoenolpyruvate), and malate synthase (Mls1p which synthesizes malate from glyoxylate). Indeed, Mdh2p, Icl1p, Pck1p, and Mls1p were in the extracellular fraction at t = 0 min and their levels were reduced at the t = 30 min time point (Figure 4A). In contrast, when cells were transferred from YPKG to YP, levels of extracellular Fbp1p, Mdh2p, Icl1p, Pck1p, and Mls1p did not decrease (Figure 4B). These results suggest that the decline of these proteins in the extracellular fraction is dependent on the presence of glucose in the media.

### Identification of extracellular proteins using iTRAQ

The findings that multiple gluconeogenic enzymes showed reduced levels in the extracellular fraction after a transfer of cells to YPD prompted us to hypothesize that changes in the secretome induced by glucose were not limited to gluconeogenic enzymes. To identify other extracellular proteins that changed levels in cells that were transferred from low to high glucose media, we used the iTRAQ technique that we previously employed to identify proteins in the total proteome that change their relative amounts under the same conditions [55]. Because a significant reduction in protein levels in the extracellular fraction was observed at the t = 30 min time point for gluconeogenic enzymes, our iTRAQ experiments were performed using wild-type cells that were transferred to YPD for 30 min. For these iTRAQ experiments, gluconeogenic enzymes that were shown to be in the extracellular space were considered as positive controls that should be identified in the extracellular fraction. Their levels should decrease in the extracellular fraction at the t = 30 min time point. Additional controls for extracellular proteins included isoforms of glyceraldehyde-3-phosphate dehydrogenase Tdh1p, Tdh2p, and Tdh3p that have been shown to be on the cell-surface of *Saccharomyces cerevisiae*[11,38,67]. Ssa1p and Ssa2p are on the cell surface of *Saccharomyces cerevisiae* as indicated by immunofluorescence microscopy [15] and served as additional controls for extracellular proteins. Invertase Suc2p and the constitutively expressed acid phosphatase Pho3p are secreted into the periplasm [68,69] and should also be identified in the extracellular fraction.

**Figure 5:**
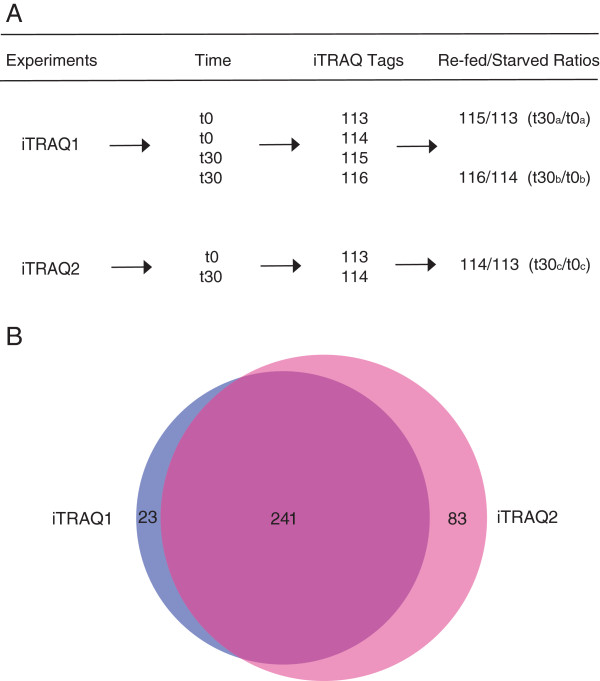
**Experimental Designs for iTRAQ1 and iTRAQ2, and the distribution of proteins identified in iTRAQ1 vs iTRAQ2. (A)** Schematic drawing of the iTRAQ1 and iTRAQ2 experiments examining changes in t30/t0 ratios. Wild-type cells were grown in YPKG or transferred to YPD for 30 minutes. Extracellular proteins were extracted from t = 0 and t = 30 cells and digested with trypsin. In iTRAQ1, peptides from replicates of t = 0 cells were labelled with 113 and 114, whereas peptides from replicates of t = 30 cells were labelled with 115 and 116. We used 115/113 (t30_a_/t0_a_) and 116/114 (t30_b_/t0_b_) to identify proteins that showed altered t30/t0 ratios. In iTRAQ2, peptides from t = 0 cells were labelled with113, whereas peptides from t = 30 cells were labelled with 114. We then used 114/113 (t30_c_/t0_c_) to identify proteins that showed changes in t30/t0 ratios. **(B)**, BioVenn analysis [100] was used to compare proteins identified in iTRAQ1 and iTRAQ2. In this current study, 241 proteins overlapped in both iTRAQ1 and iTRAQ2, whereas 23 were uniquely identified in iTRAQ1 and 83 proteins were uniquely identified in iTRAQ2.

Wild-type cells were grown in YPKG for 72 hours after which equal amounts of cells (OD_600_ =10) were either harvested at t = 0 min or transferred to YPD for 30 min (t = 30 min). Extracellular proteins were extracted from t = 0 and t = 30 cells, precipitated with TCA and digested with trypsin. The resulting tryptic peptides were then labelled with different iTRAQ tags and subjected to 2D-LC/MALDI-MS/MS (Figure 5A). In the first iTRAQ experiment (called iTRAQ1 in this study), peptides from replicates of t = 0 cells were labelled with the 113 and 114 tags, whereas peptides from replicates of t = 30 cells were labelled with 115 and 116 tags. We then used the 115/113 (t30_a_/t0_a_) and 116/114 (t30_b_/t0_b_) ratios to identify proteins that changed levels after a shift to YPD. In the iTRAQ1 experiment, Mdh2p, Icl1p, Pck1p, Mls1p, Tdh1p, Tdh2p, Tdh3p, Ssa1p, Ssa2p, Suc2p, and Pho3p were identified with multiple peptides having more than 95% confidence (Additional file 1: Table S1), although for unknown reasons, Fbp1p was not identified in the iTRAQ1 experiment. In a repeat of the same iTRAQ experiment using similar approaches (called iTRAQ2 in this study), peptides from t = 0 cells were labelled with the 113 iTRAQ tag, whereas peptides from t = 30 cells were labelled with the 114 iTRAQ tag. We then used the 114/113 (t30_c_/t0_c_) ratios to quantitate level changes in the identified proteins (Figure 5A). In the iTRAQ2 experiment, all 12 positive control proteins including Fbp1p were identified with multiple peptides having at least 95% confidence (Additional file 1: Table S1). The identification of these proteins known to be in the extracellular fraction thus validated our iTRAQ studies.

Overall, we have confidently identified 318 extracellular proteins in iTRAQ1 and 392 extracellular proteins in ITRAQ2 with an estimated local False Discovery Rate (FDR) of less than 5%. Proteins from which only a single peptide had been identified were manually inspected and only those that were identified in both experiments were included. (Proteins that were identified with a single peptide with 95% confidence in only one of the two iTRAQ experiments were not included). After removing those single-peptide proteins, a total of 347 extracellular proteins remained including 264 extracellular proteins from iTRAQ1 and 324 proteins from iTRAQ2. The name of each identified protein with their accession number and the number of peptides identified with at least 95% confidence are listed in Additional file 1: Table S1. In general, both iTRAQ1 and iTRAQ2 identified similar sets of proteins, but iTRAQ2 identified additional proteins that nonetheless belonged to the same biological functions. For instance, there are four Ssa proteins in the Hsp70 family in *Saccharomyces cerevisiae*. Three of the four Ssa proteins (Ssa1p, Ssa2p, and Ssa4p) were identified in iTRAQ1, whereas all four Ssa proteins were identified in iTRAQ2. As seen in the Venn diagram (Figure 5B), 241 identified proteins were seen in both iTRAQ experiments, whereas 23 proteins were uniquely identified in iTRAQ1 and 83 proteins were uniquely identified in iTRAQ2.

The biological functions of these proteins were further classified according to the Yeast Gene Ontology Slim Mapper and compared to the 6334 total yeast ORFs (Table 1). Again, proteins identified in both iTRAQ1 and iTRAQ2 showed similar distribution patterns in their biological functions. For example, compared to the total number of proteins in each category encoded in the yeast genome, we have identified relatively higher percentages of proteins involved in biosynthetic processes, small molecule metabolism, nitrogen compound metabolism, catabolic process, response to stress, amino acid metabolism, carbohydrate metabolism, cofactor metabolism, and protein folding. However, we identified relatively lower percentages of proteins involved in cellular component assembly, protein modification, cell cycle, transcription, and mRNA processing (Table 1).

**Table 1:** Comparison of functions of proteins identified in iTRAQ1 and iTRAQ2 to the yeast ORFs

**Go functions**	**A**		**B**		**C**	
	**iTRAQ1 (n = 264)**		**iTRAQ2 (n = 324)**		**Genome (n = 6334)**	
	**Frequency**	**%**	**Frequency**	**%**	**Frequency**	**%**
Biosynthetic process	135	50.90%	166	47.60%	2066	32.60%
Small molecule metabolic process	107	40.40%	133	38.10%	661	10.40%
Cellular nitrogen compound metabolic process	101	38.10%	126	36.10%	1847	29.20%
Catabolic process	54	20.40%	70	20.10%	663	10.50%
Response to stress	52	19.60%	67	19.20%	642	10.10%
Cellular amino acid metabolic process	51	19.20%	64	18.30%	247	3.90%
Carbohydrate metabolic process	48	18.10%	54	15.50%	268	4.20%
Transport	43	16.20%	54	15.50%	1084	17.10%
Translation	39	14.70%	45	12.90%	703	11.10%
Generation of precursor metabolites and energy	31	11.70%	35	10.00%	161	2.50%
Cofactor metabolic process	19	7.20%	24	6.90%	146	2.30%
Protein folding	17	6.40%	23	6.60%	89	1.40%
Cellular component assembly	17	6.40%	21	6.00%	598	9.40%
Cell cycle	17	6.40%	20	5.70%	531	6.30%
Homeostatic process	17	6.40%	20	5.70%	225	3.60%
Lipid metabolic process	17	6.40%	22	6.30%	295	8.40%
Cellular protein modification process	16	6.00%	21	6.00%	612	4.70%
Ribosome biogenesis	15	5.70%	18	5.20%	402	6.30%
Reproduction	15	5.70%	18	5.20%	429	6.80%
DNA metabolic process	14	5.30%	14	4.00%	455	7.20%
Biological process unknown	14	5.30%	32	9.20%	1188	18.80%
Transcription, DNA-dependent	13	4.90%	15	4.30%	640	10.10%
Sulfur compound metabolic process	13	4.90%	14	4.00%	89	1.40%
Membrane organization	12	4.50%	16	4.60%	214	3.40%
Chromosome organization	12	4.50%	15	4.30%	410	6.50%
Vesicle-mediated transport	12	4.50%	14	4.00%	346	5.50%
Cell wall organization or biogenesis	11	4.20%	13	3.70%	186	2.90%
Aging	11	4.20%	12	3.40%	70	1.10%
Cytoskeleton organization	10	3.80%	16	4.60%	220	3.50%
tRNA metabolic process	10	3.80%	14	4.00%	158	2.50%
Macromolecular complex assembly	10	3.80%	12	3.40%	421	6.60%
Protein targeting	10	3.80%	17	4.90%	262	4.10%
Cell differentiation	10	3.80%	11	3.20%	154	2.40%
Anatomical structure development	10	3.80%	11	3.20%	150	2.40%
Anatomical structure formation in morphogenesis	10	3.80%	11	3.20%	129	2.00%
Mitochondrion organization	9	3.40%	10	2.90%	339	5.40%
Nucleocytoplasmic transport	9	3.40%	8	2.30%	162	2.60%
Ribonucleoprotein complex assembly	8	3.00%	9	2.60%	130	2.10%
Signal transduction	8	3.00%	10	2.90%	162	3.50%
Growth	7	2.60%	8	2.30%	142	2.20%
Transmembrane transport	7	2.60%	8	2.30%	220	3.50%
Nucleobase-containing compound catabolic process	6	2.30%	8	2.30%	158	2.50%
Mitosis	5	1.90%	6	1.70%	119	1.90%
Chromosome segregation	5	1.90%	6	1.70%	132	2.10%
Cell death	4	1.50%	5	1.40%	25	0.40%
Cell division	4	1.50%	6	1.70%	134	2.10%
Protein complex assembly	3	1.10%	4	1.10%	221	3.50%
mRNA processing	2	0.80%	2	0.60%	192	3.00%
Cytoskeleton-dependent intracellular transport	2	0.80%	2	0.60%	19	0.30%
Cell adhesion	1	0.40%	1	0.30%	10	0.20%
Plasma membrane organization	1	0.40%	1	0.30%	6	0.10%
Vacuolar transport	1	0.40%	1	0.30%	142	2.20%
Cell morphogenesis	1	0.40%	1	0.30%	29	0.50%
Protein maturation	0	0.00%	0	0.00%	45	0.70%

Comparison of the biological functions of the proteins identified in iTRAQ1 (A) and iTRAQ2 (B) to the 6334 yeast ORFS (C) using the gene ontology (GO) annotations from the *saccharomyces* genome database.

### Classification of extracellular proteins identified by iTRAQ

According to our iTRAQ data, a large number of extracellular proteins that we have identified were involved in the metabolisms of carbohydrates, amino acids, lipids, and nucleotides (Additional file 1: Table S1). For the metabolism of carbohydrates, enzymes involved in glycolysis/gluconeogenesis, the pentose pathway, glycogen biosynthesis, the TCA cycle, and alcohol production were identified as present in the extracellular fraction (Additional file 1: Table S1). The secretion of these enzymes in response to different stimuli has been reported in many studies; for example, glycolytic enzymes are secreted as immunogens during infection by *Candida albicans*[16,38,70]*,* and GAPDH and enolase may be secreted during invasion by pathogens, as they bind to various mammalian proteins such as lysozyme, fibronectin, actin, myosin, and plasmin [16,38,70]. Elevated levels of glycolytic enzymes are secreted from various cancer cells [7,26-29,71]. Furthermore, enolase, GAPDH, 3-phosphoglycerate kinase, alcohol dehydrogenase, pyruvate kinase, fructose-1,6-bisphosphate aldolase, phosphoglycerate mutase and triose phosphate isomerase are commonly identified in the secretomes from bacteria, fungi, parasites and mammalian cells [6,7,22-24,27,37,70]. In this current study, we have also identified enzymes involved in glycolysis, the pentose pathway, glycogen biosynthesis, alcohol metabolism, as were a large number of proteins involved in the metabolisms of amino acids and purines/pyrimidines.

Anti-oxidant proteins are important for detoxifying potentially damaging reactive oxygen and nitrogen intermediates. The anti-oxidant proteins superoxide dismutase and thioredoxin peroxidase have been reported in various secretomic studies [20,27,31,36,72]. Many of these anti-oxidant proteins were identified in our study. Heat shock proteins Hsp70, Hsp90, and Hsp97 constituted the largest protein family in the secretome from adult worms of *Schistosoma japonicum*[73]. The immunodominant 47-kDa antigen in *Candida albicans* was the breakdown product of Hsp90 and was detected in patients with systemic candidiasis [38,74]. The Hsp70s play important roles in protein folding, protein translocation across membranes, gene regulation, and protection from heat shock [75,76]. Multiple protein-folding ATPase, heat shock proteins, and co-chaperones were also identified in the current study. Protein disulfide isomerases and cyclophilins may act with heat shock proteins to assist in protein folding and are frequently observed in many secretomic studies from different species [6,26,27,36]. The current study also identified proline isomerase, proline rotamase, and protein disulfide isomerase.

### Validation of extracellular proteins identified by iTRAQ

**Figure 6:**
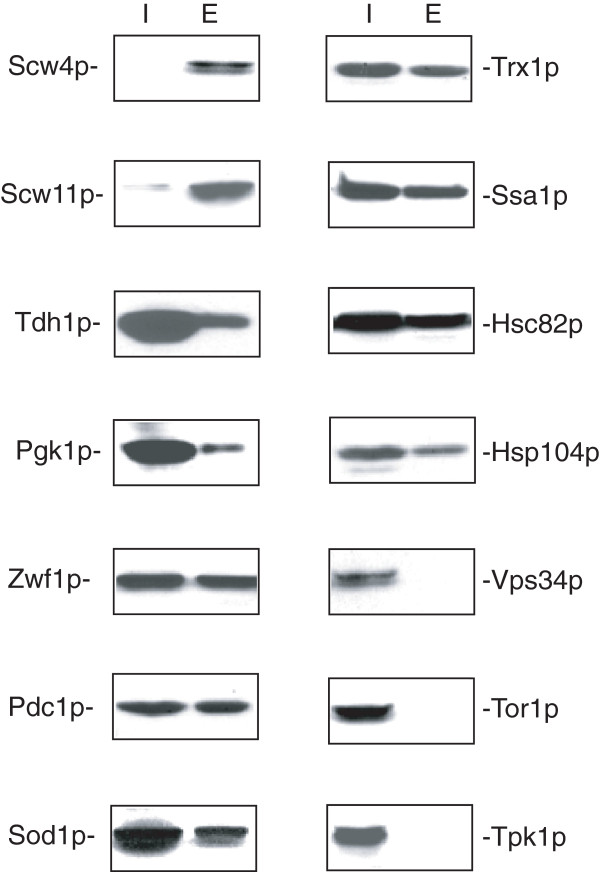
**Proteins involved in different biological functions are in the extracellular fraction in cells grown in low glucose.** Wild-type cells expressing Scw4p-GFP, Scw11p-GFP, Tdh1p-GFP, Pgk1p-GFP, Zwf1p-GFP, Pdc1p-GFP, Sod1p-GFP, Trx1p-GFP, Ssa1p-GFP, Hsc82p-GFP, Hsp104p-GFP, Vps34p-GFP, Tor1p-GFP, and Tpk1p-GFP were grown in YPKG media. The distribution of GFP tagged proteins in the intracellular (I) and the extracellular (E) fractions was examined by Western blotting using anti-GFP antibodies.

We used the extraction protocol and Western blotting to validate the extracellular location of 10 candidate proteins identified from the iTRAQ experiments (Figure 6). Wild-type cells expressing GFP-tagged proteins were grown in YPKG and subjected to the extraction procedure. Levels of GFP tagged proteins in the intracellular and extracellular fractions were examined using anti-GFP antibodies. To rule out the possibility that GFP tagging caused non-specific appearance of proteins in the extracellular fraction, we first examined the distribution of proteins known to be in the intracellular fraction that were fused with GFP. For example, signalling molecules involved in the inactivation and degradation of gluconeogenic enzymes are distributed mostly in the intracellular fraction under the same experimental conditions [77]. Vps34p is the major phosphatidylinositol 3-kinase in yeast [78] and is involved in the degradation of Fbp1p [77]. Tor1p is a subunit of the TORC1 complex [79] required for the degradation of gluconeogenic enzymes [66]. Tpk1p is a subunit of the protein kinase A that phosphorylates Fbp1p in response to glucose replenishment [57-59]. GFP fusion to these proteins should not change the distribution of these proteins in the intracellular fraction.

We next confirmed that proteins that are known to be present in the extracellular fraction/cell-surface were indeed distributed in the extracellular fraction. Scw4p and Scw11p are soluble cell wall proteins [80], and the majority of Scw4p and Scw11p was found in the extracellular fraction (Figure 6). We then determined the distribution of enzymes involved in carbohydrate metabolism. Tdh1p is a cell-surface protein [11,38,67], and was in the extracellular fraction (Figure 6). Pgk1p is 3-phosphoglycerate kinase that transfers high-energy phosphoryl groups from 1,3-bisphosphoglycerate to ADP during glycolysis and the reverse reaction during gluconeogenesis [81]. Zwf1p is glucose-6-phosphate dehydrogenase that catalyzes the first and irreversible step of the pentose phosphate pathway [82]. Pdc1p is the major pyruvate decarboxylase that decarboxylates pyruvate to acetaldehyde for ethanol production [83]. Consistent with our iTRAQ results, Pgk1p, Pdc1p, and Zwf1p were detectable in the extracellular fraction (Figure 6). We also examined the distribution of proteins involved in oxidative stress. Sod1p is the copper-zinc superoxide dismutase that detoxifies superoxide [84], while Trx1p is thioredoxin that protects cells against oxidative and reductive stress [85]. Both Sod1p and Trx1p were in the extracellular fraction (Figure 6). Finally, we examined the distribution of heat shock proteins. As mentioned above, Ssa1p is a cell-surface protein [15] and was in the extracellular fraction (Figure 6). Hsc82p is required for folding of nascent polypeptides as well as for the refolding of denatured proteins [86]. Hsp104p refolds and reactivates previously denatured and aggregated proteins [87]. Hsc82p and Hsp104p were also present in the extracellular fraction (Figure 6). In contrast, Vps34p, Tor1p, and Tpk1p were distributed mostly in the intracellular fraction (Figure 6). Thus, proteins participate in many different biological functions including those involved in glycolysis/gluconeogenesis, the pentose pathway, alcohol production, oxidative stress, and protein folding are all present in the extracellular faction.

### Validation of the t30/t0 ratios

We next used the t30_a_/t0_a_ and t30_b_/t0_b_ ratios from iTRAQ1 and the t30_c_/t0_c_ ratio from iTRAQ2 to identify proteins that changed levels following a transfer to YPD (Additional file 2: Table S2). The Protein Pilot Program calculates a p-value for each protein t30/t0 ratio based on the mean of the t30/t0 ratios of all the peptides belonging to that protein, and the scatter of those peptide ratios. We highlighted in bold the protein t30/t0 ratio whose calculated p-value was less than 0.05 (Additional file 2: Table S2). Also highlighted (Additional file 2: Table S2) are a total of 230 proteins that contained at least one of the three t30/t0 ratios having p-values smaller than 0.05. Because gluconeogenic enzymes decrease their levels after cells are transferred to YPD, they should have reduced t30/t0 ratios. Consistent with this idea, the t30/t0 ratios for these gluconeogenic enzymes were reduced. In a similar manner, significantly decreased t30/t0 ratios were observed for enzymes involved in glycolysis, the pentose pathway, alcohol production, glycogen biosynthesis, and the TCA cycle. Moreover, proteins involved in oxidative stress, in protein folding, and in other functions also exhibited a significant reduction in t30/t0 ratios (Additional file 2: Table S2).

**Figure 7:**
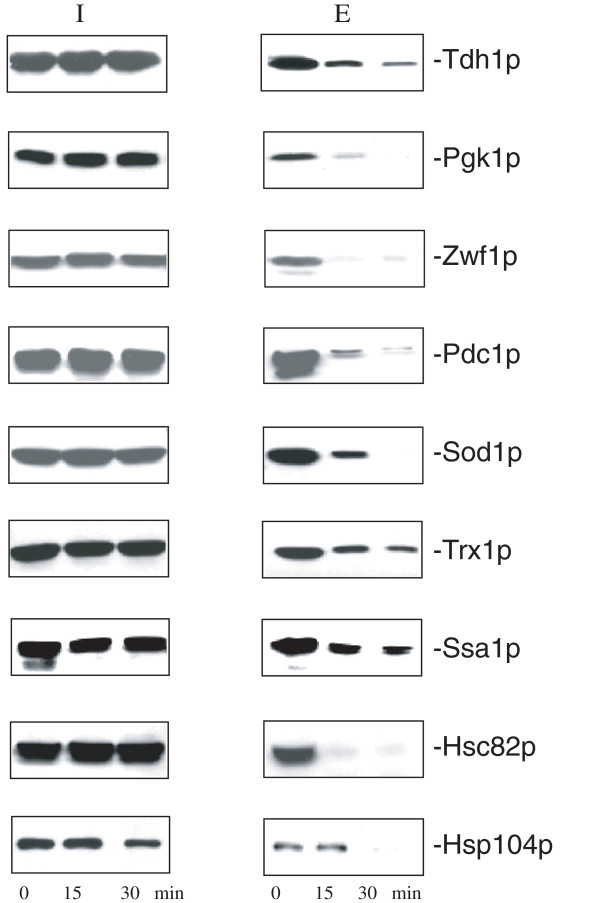
**Levels of extracellular proteins involved in different functions decrease upon a transfer from YPKG to YPD.** Wild-type cells expressing Tdh1p-GFP, Pgk1p-GFP, Zwf1p-GFP, Pdc1p-GFP, Sod1p-GFP, Trx1p-GFP, Ssa1p-GFP, Hsc82p-GFP, and Hsp104p-GFP were transferred from YPKG to YPD for 0, 15, and 30 min. The distribution of GFP tagged proteins in the intracellular and extracellular fractions was examined by Western blotting.

According to our iTRAQ data, t30/t0 ratios were reduced for Tdh1p, Pgk1p, Zwf1p, and Pdc1p (Additional file 2: Table S2). Our iTRAQ results also indicated that Sod1p, Trx1p, Ssa1p, Hsc82p, and Hsp104p decreased their t30/t0 ratios following a transfer of cells to YPD. To confirm that these extracellular proteins reduced their levels following glucose addition in YPD, wild-type cells expressing GFP tagged proteins were grown in YPKG media and transferred to YPD for 0, 15, and 30 min. Cells were subjected to the extraction procedure and the distribution of GFP-tagged proteins in the intracellular and extracellular fractions was determined by Western blotting using anti-GFP antibodies (Figure 7). At t = 0 min, Tdh1p, Pgk1p, Zwf1p, and Pdc1p were detectable in the extracellular fraction. Following a transfer of cells from YPKG to YPD for up to 30 min, their levels in the extracellular fraction decreased rapidly. In a similar manner, Sod1p, Trx1p, Ssa1p, Hsc82p, and Hsp104p were in the extracellular fraction when cells were grown in YPKG. Levels of these proteins in the extracellular fraction decreased after a shift of cells to YPD for 30 min. Taken together, these proteins that are involved in different biological functions show very similar distribution characteristics. They are all present in the extracellular fraction during growth in YPKG and their levels in the extracellular fraction are all reduced following a transfer of cells to YPD for 30 min.

### Small Vesicles disappear in response to glucose re-feeding

**Figure 8:**
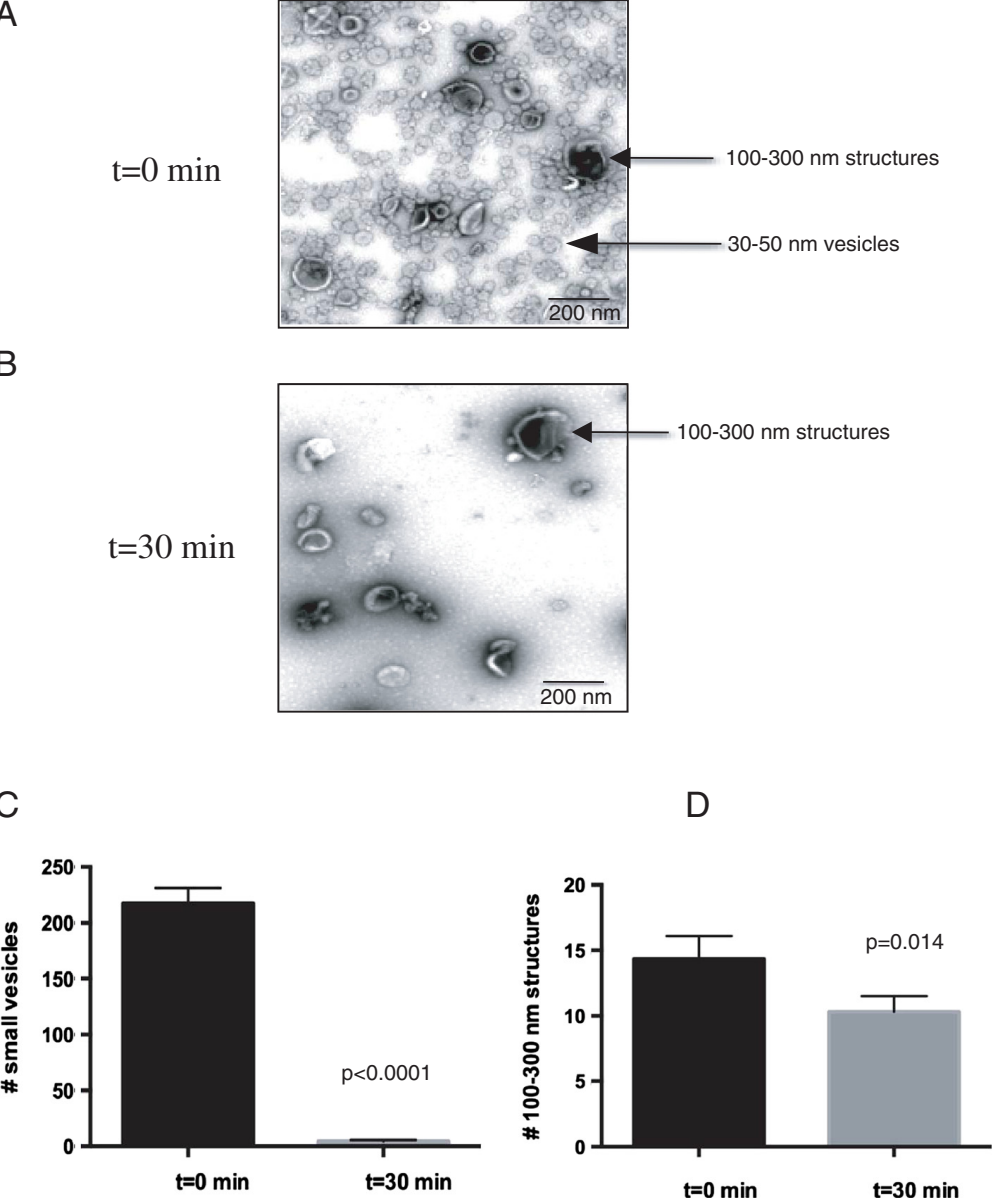
**Transferring cells from YPKG to YPD causes a rapid disappearance of small vesicles from the extracellular fraction. (A and B)** Wild-type cells were grown in YPKG or transferred to YPD for 30 minutes and extracted. Total extracts from t = 0 **(A)** and t = 30 **(B)** cells were stained with uranyl acetate and visualized by transmission electron microscope as described in Methods. Bars: 200 nm. **(C)** Quantification of the number of 30–50 nm small vesicles per μm^2^ in total extracts in t = 0 and t = 30 cells. Mean and SD were derived from counting the number of small vesicles using 3 images taken from extracts isolated from t = 0 and t = 30 cells. **(D)** Quantification of the number of 100–300 nm structures per μm^2^ in total extracts in t = 0 and t = 30 cells. Mean and SD were obtained from counting the number of 100–300 nm structures from 3 images taken from total extracts isolated from t = 0 and t = 30 cells.

The extraction procedure employed in the current work has been used to study aspects of protein composition in the extracellular fraction [2,64,77]. However, the contents of the extracted materials had not previously been visualized at the ultra-structural level. Small vesicles called exosomes are released from a variety of mammalian cells. In yeast, Fbp1p is associated with small vesicles that are 30–50 nm in diameter [88]. Because of the size of these vesicles, they can only be visualized by EM techniques. We thus performed TEM studies to examine whether or not small vesicles were present in the extracellular fraction in cells grown in YPKG. Wild-type cells were grown in YPKG for 3d and harvested (t = 0 min), or transferred to YPD for 30 min (t = 30 min) and subjected to the same extraction procedure. Extracted materials were centrifuged at 3,000 × g and then at 200,000 × g. The 200,000 × g pellets from the t = 0 and t = 30 cells were stained with uranyl acetate followed by visualization using TEM (Figure 8A and 8B). When total extracts were prepared from t = 0 cells, at least two types of structures were observed. Small vesicles of 30–50 nm in diameter and large structures of 100–300 nm in diameter were identified (Figure 8A, arrows). Quantification of these structures indicated that total extracts from cells grown in YPKG consisted of approximately 93.9% small vesicles and 6.1% large structures. However, when total extracts were isolated from t = 30 cells, very few small vesicles were observed, whereas the 100–300 nm large structures still remained (Figure 8B, arrows). The number of small vesicles in total extracts was 218.3 ± 13.5 per μm^2^ at t = 0 min and was 4.5 ±1.3 per μm^2^ at t = 30 min (Figure 8C). The number of 100–300 nm large structures was 14.3 ± 1.7 per μm^2^ at t = 0 min and was 10.3 ±1.1 per μm^2^ at t = 30 min (Figure 8D). Therefore, approximately 98% of the small vesicles disappeared within 30 min following glucose replenishment in YPD. In contrast, only about 28% of the large structures had disappeared at the 30 min time point. This rapid disappearance of small vesicles from the extracellular fraction at t = 30 min demonstrates another example of rapid changes in the secretome/extracellular fraction during transition from glucose-deficient to glucose-rich media.

**Figure 9:**
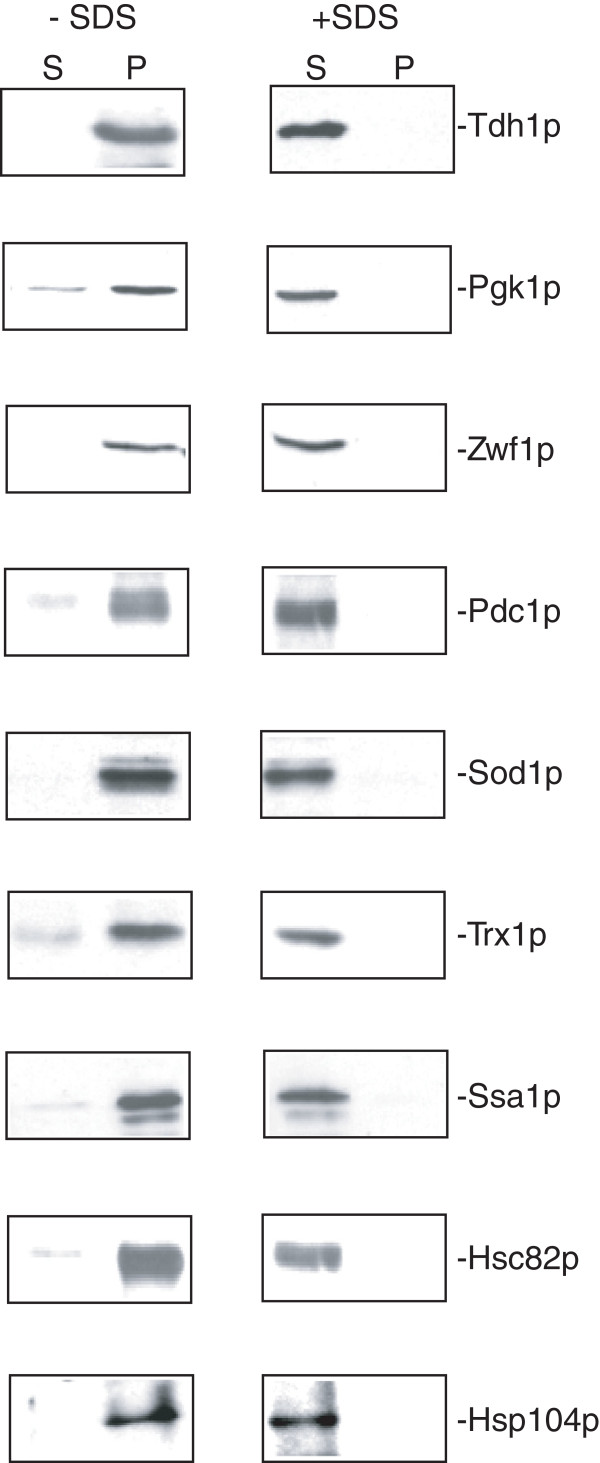
**Extracellular proteins involved in different functions are present in the vesicle-enriched fraction.** Wild-type cells expressing GFP tagged proteins were grown in YPKG for 3d and extracted. Total extracts were centrifuged first at 3,000 × g for 10 min and 200,000 × g for 2 hours. The 200,000 × g pellet fraction was resuspended in PBS and incubated in the absence or presence of 2% SDS for 30 min. Following incubation, samples were re-centrifuged at 200,000 × g for 2 hours. The distribution of proteins in the 200,000 × g supernatant (S) and pellet (P) fractions was determined by Western blotting.

Finally, we determined whether or not these extracellular proteins were distributed in the vesicle-enriched fraction. Wild-type cells expressing GFP fusion proteins were grown in YPKG for 3d and extracted. Total extracts were first centrifuged at 3,000 × g and then at 200,000 × g. The 200,000 × g pellet fraction was resuspended in PBS and incubated in the absence or presence of 2% SDS for 30 min. Following incubation, samples were re-centrifuged at 200,000 × g for 2 hours and the distribution of proteins in the 200,000 × g supernatant (S) and pellet (P) fractions was then examined. In the absence of detergent, most of the proteins were in the 200,000 × g pellet fraction (Figure 9, left panels), suggesting that these proteins are associated with the small vesicles. However, when membranes were disrupted by SDS, the majority of these proteins were in the 200,000 × g supernatant fraction (Figure 9, right panels). Thus, the distribution of these proteins in the vesicle-enriched fraction is dependent on membrane integrity.

## Discussion

**Figure 10:**
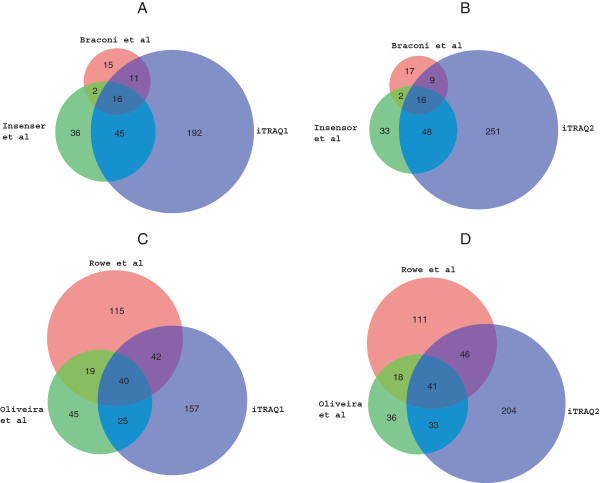
**Comparison of proteins identified in our study and the studies by Braconi et al., Insenser et al., Rowe et al., and Oliveira et al.** Proteins that were identified by Braconi et al. and Insenser et al. were compared with those identified in iTRAQ1 **(A)** and iTRAQ2 **(B)**. Proteins identified by Rowe et al. and Oliveira et al. were compared with those identified in iTRAQ1 **(C)** and iTRAQ2 **(D)**.

We performed two iTRAQ experiments and identified a total of 347 proteins that were present in the extracellular fraction in wild-type cells that were grown in YPKG. The vast majority of the proteins identified in this study lack typical ER signal sequence, suggesting that they are secreted via the non-classical pathway. A small number of proteins identified in the current study, including Ape2p, Bgl2p, Exg1p, Scw4p, Suc2p, Pep4p, Prb1p, Prc1p, Pho3p, Pho5p, Pho12p, Ygp1p, and Uth1p, did however contain ER-signal peptides and were thus presumably secreted by the classical pathway. Our observations of the relatively low number of classic pathway secreted proteins are consistent with another secretomic/surfomic study in *Saccharomyces cerevisiae* in which only 17 proteins of a total of 99 proteins contained a signal sequence [72]. When we compared our iTRAQ1 results with two previous *Saccharomyces cerevisiae* secretomic/surfomic studies [17,72], 16 proteins overlapped in all 3 studies (Figure 10A). Overlapping proteins included Eno1p, Eno2p, Hxk2p, Pgk1p, Tdh3p, Adh1p, Pdc1p, Hsp82p, Ssa1p, Ssa2p, Ssb1p, Sse1p, Sod1p, Act1p, Bmh1p, and Exg1p. As indicated in the Venn diagram (Figure 10A), 192 proteins were unique to iTRAQ1, whereas 36 proteins were unique to the study by Insenser et al. [72] and 15 proteins by Braconi et al. [17]. We obtained very similar distribution patterns when we compared iTRAQ2 with the studies by Braconi et al. and Insenser et al. (Figure 10B), where the same 16 proteins overlapped. The observed low numbers of overlapping proteins may result from variations in the number of proteins identified in each study. When we compared the proteins we identified with those identified by Rowe et al. [89], 82 proteins overlapped with iTRAQ1 (Figure 10C) and 87 proteins overlapped with iTRAQ2 (Figure 10D). Common proteins included metabolic enzymes, heat shock proteins and proteins involved in oxidative stress, protein folding, and protein translation. Since we used cells that were grown in low glucose, whereas other studies used cells grown in high glucose, the identification of these common proteins in the secretome appears to be independent of the growth conditions. Furthermore, because different methods such as trypsin shaving, biotinylation or extraction with reducing agents were utilized in different studies, these proteins are unlikely to be released into the extracellular fraction due to cell lysis. In our study, both extracted and non-extracted cells were not stained with trypan blue, suggesting that cell lysis is minimal. In contrast, when Triton-X100 was added to the extracted cells and then incubated with trypan blue, high percentages of cells were stained. It was also observed that non-extracted and extracted cells were able to internalize exogenously added FM dye and actively transport it to the vacuole, suggesting that the endocytic pathway is functional in these cells.

In this study, we used wild-type cells that were grown in YNB-based or YPKG-based media to examine glucose effects in up-regulating Lia1p and down-regulating Fbp1p. Amounts of Lia1p and Fbp1p were low in cells grown in YNB-based media and were higher in cells grown in YPKG. Adding glucose directly to the existing YPKG media produced a slow down-regulation of Fbp1p, whereas up-regulation of Lia1p was not observed. One possible explanation for these results is that metabolites or wastes present in the existing media prevented a fast increase in Lia1p levels and a fast decrease in Fbp1p levels. In contrast, changing the culture media from YPKG to YPD produced a faster down-regulation of Fbp1p and a faster up-regulation of Lia1p. Therefore, the YPKG to YPD condition is appropriate to study glucose effects in regulating protein levels. Down-regulation of gluconeogenic enzymes under this experimental condition is caused by degradation of pre-existing proteins in the vacuole. In contrast, when cells were grown in the presence of high glucose for a prolonged period of time, gluconeogenic enzymes are either not expressed or expressed at low levels [90-94]. Low expression levels of these enzymes are likely a result from low rates of protein synthesis. Therefore, the underlying mechanisms for catabolite inactivation of gluconeogenic enzymes in cells that are transferred from YPKG to YPD are fundamentally different from low rates of synthesis of these enzymes in cells that are continuously grown in the presence of high glucose.

It is interesting that proteins that are involved in so many diverse functions are secreted from cells. The secretion of these metabolic enzymes may be dependent on the needs of cells. For example, metabolic enzymes may be secreted when the needs inside the cells are decreased. If this is true, the extracellular space might be used as a storage place for these proteins. It is possible that secreted proteins have functions outside the cells similar to what they normally perform inside the cells. Alternatively, these proteins may have functions in the extracellular fraction unrelated to their functions in metabolism. For example, GAPDH and enolase bind to components of the extracellular matrix and are involved in the invasion of *Candida albicans* into host tissues [14,38].

Our TEM studies indicated that small vesicles were present in the extracellular fraction in cells grown in YPKG and that they disappeared following a shift to YPD. A rapid disappearance of small vesicles from the extracellular fraction at the t = 30 time point may be caused by internalization into the cytoplasm or release into the culture media. The presence of extracellular vesicles in the culture media in *Saccharomyces cerevisiae* has been documented by Oliveira et al. [95]. Interestingly, 82 proteins identified in extracellular vesicles isolated from the culture media overlapped with the proteins that we identified in iTRAQ1 (Figure 10C) and 87 proteins overlapped with iTRAQ2 (Figure 10D). In our study, cells were harvested by a low speed centrifugation and the culture media was discarded following centrifugation. The extraction procedure was then performed on cells that were pelleted. Therefore, the proteins that we identified are unlikely to be derived from the culture media.

Using the extraction procedure and Western blotting, we confirmed that extracellular proteins including Tdh1p, Pgk1p, Zwf1p, Pdc1p, Sod1p, Trx1p, Ssa1p, Hsc82p, and Hsp104p were in the extracellular fraction in cells grown in YPKG. All of these proteins showed reduced t30/t0 ratios in the iTRAQ experiments, suggesting that these proteins are cleared from the extracellular fraction at the t = 30 min time point, and the extraction protocol and Western blotting confirmed that levels of these extracellular proteins decreased following a transfer of cells to YPD for 30 min. These extracellular proteins were associated with membranes in the vesicle-enriched fraction. However, when membranes were disrupted by SDS, these proteins were in the 200,000 × g supernatant fraction, suggesting that the distribution of these proteins in the vesicle-enriched fraction is dependent on membrane integrity. There are several possible paths that might be involved in the observed decreases in extracellular proteins and vesicles upon a shift of cells to high glucose, such as internalization into the cytoplasm, degradation in the extracellular space, or release into the culture media. Future experiments will be needed to elucidate the molecular mechanisms responsible for the decline of protein levels in the extracellular fraction and the disappearance of small vesicles from the extracellular fraction.

## Conclusions

Using the iTRAQ technique, we identified 347 proteins that were present in the extracellular fraction. The majority of these extracellular proteins do not contain ER signal sequences, suggesting that they are secreted via a non-classical pathway. Using the extraction procedure and Western blotting, we confirmed that enzymes involved in glycolysis/gluconeogenesis, the pentose pathway, alcohol production, oxidative stress, and protein folding were in the extracellular fraction. Furthermore, their levels were all reduced in response to changes to glucose enriched media. Our TEM studies indicated that there was a rapid disappearance of small vesicles from the extracellular fraction at the t = 30 min time point. We also observed a rapid decline in protein levels for a large number of extracellular proteins upon a transfer of cells to YPD for 30 min. We suggest that the secretome undergoes dynamic changes during transition from glucose-deficient to glucose-rich media. The plasticity of the secretome may be required to maintain the function and integrity of the cell depending on its physiological or nutritional states.

## Methods

### Cell culture, media and antibodies

Wild-type (BY4742, *MATα his3Δ1 leu2Δ0 lys2Δ0 ura3Δ0*) and wild-type cells expressing GFP tagged proteins were inoculated from -70°C frozen stock into 2 ml YPD (1% yeast extract, 2% peptone, and 2% glucose) in sterile glass tubes at 30°C in an environmental shaker at 250–300 rpm for 16 hours. Cell densities were measured using a Beckman DU640B spectrophotometer. Cells (50 μl) were diluted to OD_600_ = 0.01/ml in 10 ml YNB (0.67% yeast nitrogen base with amino acids and 2% glucose) or in 10 ml YPKG (1% yeast extract, 2% peptone, 1% potassium acetate, and 0.5% glucose), in sterile glass tubes for 72 hours at 30°C until cell density reached OD_600_ = 4-5/ml. The YNB media was supplemented with 0.1 ml of the 100 × of solution containing 200 mg uracil, 200 mg L-histidine, 300 mg L-leucine, and 300 mg L-lysine. Cells containing OD_600_ = 10/ml were aliquoted and used for each time point. In experiment I, 2% glucose was added directly to the existing YNB culture and incubated for 0, 2, and 4 hours at 30°C. In experiment II, cells were pelleted by a low speed centrifugation at 3,000 × g for 10 min and resuspended in fresh YNB-based media containing 2% glucose for 0, 2, and 4 hours at 30°C. In experiment III, 2% glucose was added directly to the existing YPKG culture for 0, 2, and 4 hours at 30°C. In experiment IV, cells were pelleted and resuspended in YPD containing 2% glucose for 0, 2, and 4 hours at 30°C. Cells grown in YPKG also pelleted and resuspended in YP (without added glucose) for 0, 2, and 4 hours at 30°C. Cells (OD_600_ = 10/ml) were harvested at each time point and processed. Levels of Lia1p and Fbp1p, and Tpi1p were examined by Western blotting. For re-growth experiments, wild-type cells were grown in 10 ml YPKG for 3d at 30°C in an environmental shaker until OD_600_ = 4-5/ml. Cells were pelleted and diluted to OD_600_ = 0.2/ml in 10 ml YPD (1% yeast extract, 2% peptone, 2% glucose), 10 ml YPKG (1% yeast extract, 2% peptone, 1% potassium acetate, and 0.5% glucose) or 10 ml YP (1% yeast extract, 2% peptone) at 30°C. Cell densities were measured at OD_600_ for 0, 2, 4, 6, and 8 hours using a Beckman DU640B spectrophotometer.

### Extraction and Western blotting

Extraction of extracellular proteins was performed as described [2,64,77]. Wild-type cells and wild-type cells expressing GFP tagged proteins were inoculated from -70°C frozen stock into 2 ml YPD culture in sterile glass tubes in an environmental shaker at 250–300 rpm at 30°C for 16 hours. Cells (50 μl) were cultured into 10 ml of YPKG for 72 hours at 30°C. Cell densities were measured and cells (OD_600_ = 10/ml) were harvested (t = 0) or transferred to YPD containing glucose in an environmental shaker at 250–300 rpm at 30°C for 15, and 30 min (t = 15 and t = 30). At each time point, 10 mM NaN_3_ (final concentration) was added to stop the reactions. Cells were harvested by a low speed centrifugation at 3,000 × g for 10 minutes. Following centrifugation, the culture media was removed and cell pellets were resuspended in 100 μl of extraction buffer that contained 0.1 M Tris pH 9.4 and 10 mM β-mercaptoethanol. Samples were placed in a 37°C water bath for 15 min at 200 rpm. Following the extraction procedure, cells were pelleted at 3,000 × g for 10 minutes. The extracellular proteins (100 μl) that were released into the supernatants were precipitated in 15% trichloroacetic acid (TCA), washed, and solubilized in SDS-PAGE sample buffer. The remaining cell-associated fraction was lysed and solubilized in SDS-PAGE sample buffer. The distribution of intracellular and extracellular proteins was examined by Western blotting using Western Lighting Plus ECL kit (Perkin Elmer). Wild-type cells expressing GFP tagged proteins were purchased from Invitrogen. Anti-GFP antibodies were purchased from Abcam. Anti-TPI antibodies were from Proteintech Group.

### Trpan blue staining and FM4-64 uptake

Wild-type cells were grown in 10 ml YPKG for 3d at 30°C in an environmental shaker. Cells were either harvested at t = 0 min or transferred to YPD for 30 min at 30°C. Cells were resuspended in 200 μl PBS buffer containing 140 mM NaCl, 2.7 mM KCl, 10 mM Na_2_HPO_4_, 1.8 mM KH_2_PO_4_, pH 7.4 and incubated in the absence or presence of 2% Triton X-100 for 30 min. This was followed by incubation with 20 μl of 0.4% trypan blue in PBS for 30 min at room temperature. The same amounts of t = 0 and t = 30 cells were subjected to the extraction procedure and pelleted. Extracted cells were resuspended in 200 μl PBS and incubated in the absence or presence of 2% Triton X-100 for 30 min. Treated and non-treated cells were then incubated with 20 μl of 0.4% trypan blue for 30 min. Cells were observed using a Zeiss microscope.

For FM uptake experiments, wild-type cells were grown in 10 ml YPKG for 3d at 30°C. Cells were either harvested at t = 0 min or transferred to YPD media for 30 min (t = 30) at 30°C. Half of the cells were pelleted, resuspended in 200 μl PBS, and incubated with 1 μl FM (16 mg/ml) for 3 hours at 30°C. Another half of the cells were extracted, resuspended in 200 μl PBS and then incubated with 1 μl FM for 3 hours at 30°C. The distribution of FM was visualized using a Zeiss fluorescence microscope.

### iTRAQ labelling and data analysis

Two TRAQ labelling procedures were performed following the instructions from Applied Biosystems with minor modifications. Wild-type yeast cells were grown in YPKG and either harvested (t = 0 min) or the same amounts of cells (OD_600_ =10/ml) were transferred to media containing 2% glucose for 30 min (t = 30 min). Extracellular proteins were extracted as described above and then precipitated in 15% TCA. Protein concentrations were determined using Bio-Rad DC assay. Extracted proteins were reduced with 5 mM of tris(2-carboxyethyl) phosphine at 60°C for 60 min, and alkylated with 4 mM iodoacetamide for 30 min at room temperature in the dark. Samples were digested with 10 μg of trypsin at 48°C for 16 hours. Peptides were labelled with iTRAQ tags, combined, dried and resuspended in 500 μl of 10 mM ammonium formate, pH 3.6, in 20% acetonitrile/80% water. Labelled peptides were subjected to SCX separation by loading onto a passivated Waters 600E HPLC system with a 4.6 × 250 mm PolySULFOETHYL Aspartamide^TM^ column (PolyLC, Columbia MD) followed by elution at a flow rate of 1 ml per minute with a 1 hour gradient going from 10 mM→666 mM ammonium formate in 20% acetonitrile, pH 3.6. After evaporating and resuspending the resulting 15 SCX fractions 3 times in a SpeedVac (final resuspension in 9 ml of 2% acetonitrile, 0.1% TFA), reverse phase C18 nanoflow-LC separation of the 15 resuspended SCX fractions was accomplished by loading each fraction onto a Chromolith CapRod column (150 × 0.1 mm, Merck) from a 5 ml sample loop on an LC-Tempo MALDI separation/plate spotting system at 2 ml per minute in 6.8% acetonitrile, 0.1% TFA, then eluting with a 50 minute gradient from 6.8% to 79% acetonitrile, 0.1% TFA at a flow rate of 2.5 ml per minute. A 3 ml per minute flow of MALDI matrix solution was added in-line post-column (7 mg/ml recrystallized CHCA (a-cyano-hydroxycinnamic acid), 2 mg/ml ammonium phosphate, 0.1% trifluoroacetic acid, 80% acetonitrile), and the resulting eluant was automatically spotted onto a stainless steel MALDI target plate every 6 seconds (0.55 μl per spot), for a total of 370 spots per original SCX fraction. Each target plate was analyzed in a data-dependent manner on either an ABI 5800 MALDI TOF-TOF or an ABI 4800 MALDI TOF-TOF. The MS spectra for an entire 2D-LD experiment were thus taken from 5500 MALDI spots, averaging 500 laser shots per spot at laser power 3400. In a data-dependent manner, 10,000-15,000 MS/MS spectra were taken of the strongest example of each unique m/z mass observed across each target plate, using up to 2500 laser shots per MS/MS spectrum at laser power 4100, with CID gas at 1.2 to 1.3 × 10^-6^ Torr.

Protein identification was accomplished using the Paragon Algorithm [96] as implemented in ProteinPilot^TM^ 4.0 software (ABSciex). Search parameters were set as cysteine alkylation: iodoacetamide, ID focus: biological modifications, search effort: thorough. Combined spectra were searched against the species-specific (*S. cerevisiae*) NCBI RefSeq database concatenated with a reversed “decoy” version of the same database plus 156 common lab contaminants as described by Keller et al. [97]. To reduce the potential number of false positive protein identifications, the Paragon Algorithm search results were further filtered through the use of a very stringent Local False Discovery Rate (FDR) estimation calculated from the Proteomics System Performance Evaluation Pipeline (PSPEP) [98,99]. We accepted protein IDs with an estimated local False Discovery Rate (FDR) estimate of <5%. A total of 318 extracellular proteins were identified in iTRAQ1 (Additional files 3 and 4) and 392 extracellular proteins were identified in iTRAQ2 (Additional files 5 and 6) using these criteria for acceptance. We next removed proteins that were identified by only a single confidently identified peptide in only one of the two iTRAQ experiments, (proteins that were identified with a single peptide with 95% confidence, but in both experiments, were included). From the identifiers (gi numbers) obtained from searching against the NCBI RefSeq database, we used the *Saccharomyces* Genome Database (Stanford University) and the Gene Ontology Slim Mapper to search for the function for each protein. BioVenn analysis was used to compare proteins identified from different studies [100].

### Transmission electron microscopy

Immuno-TEM was performed as described previously [77,101]. Wild-type cells were grown in 2 ml YPKG for 3d and harvested (t = 0 min) or transferred to YPD for 30 min and harvested (t = 30 min). The cells were fixed with 3% para-formaldehyde and 0.2% glutaraldehyde for 16 hours. Cells were then washed by centrifugation at 4,500 × g sequentially in 1 ml of 0.5 M sorbitol in 0.08 M potassium phosphate buffer (pH 6.7), 0.25 M sorbitol in 0.08 M potassium phosphate buffer (pH 6.7), and 0.08 M potassium phosphate buffer (pH 6.7). Cells were resuspended in 1 ml of sodium metaperiodate (1% w/v), incubated at room temperature for 20 min, re-suspended in 1 ml of 50 mM ammonium chloride, and then incubated for 15 min at room temperature. Cells were centrifuged at 4,500 × g and dehydrated by 5 min incubation in 1 ml 50% cold ethanol, 70% cold ethanol and 80% cold ethanol followed by 10 min incubation in 85% cold ethanol, 90% cold ethanol, 95% cold ethanol and 100% cold ethanol. Finally, 1 ml 100% ethanol was added to the cell pellet and incubated for 5 min at room temperature. After dehydration, cells were sequentially incubated in 1 ml LR White 2:1, ethanol:LR White at room temperature rotator for 2 hours, 1:1 ethanol:LR White resin at room temperature rotator overnight, 1:2 ethanol:LR White resin at room temperature rotator for 2 hours and 100% resin at room temperature rotator for 2 hours three times. Cells were transferred to gelatin capsules, dried and cut into 10 nm thin sections. Sections were placed onto grids and incubated with affinity purified Fbp1p antibodies followed by goat anti-rabbit antibodies conjugated with 10 nm gold particles (Ted Pella) and viewed on a JEOL JEM-1400 electron microscope.

For negative staining and TEM, wild-type cells were grown in 200 ml of YPKG in sterile glass flasks for 72 hours and split into equal aliquots based on cell density. Cells (100 ml) were transferred to YPD media containing 2% glucose for 30 minutes (t = 30 min). Cells (100 ml) from the t = 0 and t = 30 time points were harvested and resuspended in 4 ml extraction buffer containing 0.1 M Tris pH 9.4 and 10 mM β-mercaptoethanol at 37°C for 15 minutes. Total extracts from the t = 0 and t = 30 cells were centrifuged at 3,000 × g for 10 minutes at 4°C. The 3,000 × g supernatant was further centrifuged at 200,000 × g for 2 hours at 4°C. The resulting 200,000 × g pellets were resuspended in 30 μl of PBS. Samples were fixed with 3% para-formaldehyde and 0.2% glutaraldehyde for two hours at 4°C. Nickel grids were incubated with 10 μl of poly-lysine buffer for 5 minutes and washed with distilled water for 3 minutes. Total extracts (10 μl) were incubated with poly-lysine coated grids for 30 minutes. The grids were blotted with 0.1% BSA in PBS buffer. The grids were washed once with PBS buffer and twice with distilled water. The grids were then blotted with Whatman filter papers and stained with 2% uranyl acetate for 10 minutes in the dark. The grids were dried by blotting with Whatman filter papers and samples were viewed on a JEOL JEM-1400 electron microscope.

## Competing interests

The authors declare that they have no competing interests.

## Authors’ contributions

BJG participated in sample preparations, data collection, and analysis. BAS was involved in data interpretation and drafting of this manuscript. HLC was involved in the experimental design and drafting of this manuscript. All three authors read the article and approved the authorship. All authors read and approved the final manuscript.

## Supplementary Material

Additional file 1: Table S1 Total proteins identified in this study. Click here for file

Additional file 2: Table S2 Proteins that showed altered re-fed/starved ratios. Click here for file

Additional file 3 Proteins identified with a local FDR <5% in iTRAQ1. Click here for file

Additional file 4 Peptide summary information for iTRAQ1. Click here for file

Additional file 5 Proteins identified with a local FDR <5% in iTRAQ2. Click here for file

Additional file 6 Peptide summary information for iTRAQ2. Click here for file
