# Supplementary material for: Glucose induces rapid changes in the secretome of Saccharomyces cerevisiae
Source: Proteome Sci. 2014 Feb 12;12:9. doi: 10.1186/1477-5956-12-9 (PMC3927832; doi:10.1186/1477-5956-12-9)
Supplement: Additional file 2: Table S2 — Proteins that showed altered re-fed/starved ratios. [file 1477-5956-12-9-S2.doc]

**Additional file 2: Table S2. Proteins that showed altered Re-fed/Starved ratios.**

| **Table 2 Proteins that showed altered re-fed/starved ratios** | | | | | |  |  |
| --- | --- | --- | --- | --- | --- | --- | --- |
|  |  | |  | |  |  |  |
|  | **t30_a_/t0_a_** | |  | | **t30_b_/t0_b_** |  | **t30_c_/t0_c_** |
| **A. Carbohydrate Metabolism** |  | |  | |  |  |  |
| **Aco1p, Aconitase** | **0.419** | |  | | 0.651 |  | 0.301 |
| **Adh1p, Alcohol dehydrogenase** | **0.275** | |  | | **0.416** |  | **0.163** |
| **Adh2p, Glucose-repressible alcohol dehydrogenase II** | | |  | |  |  | **0.178** |
| **Adh6p, Alcohol dehydrogenase** | **0.302** | |  | | **0.453** |  | 0.318 |
| **Ald3p, Aldehyde dehydrogenase** |  | |  | |  |  | **0.217** |
| **Ald6p, Aldehyde dehydrogenase** | **0.361** | |  | | **0.455** |  | **0.250** |
| **Ara1p, NADP+ dependent arabinose dehydrogenase** | **0.354** | |  | | **0.578** |  | **0.232** |
| **Atp2p, ATP synthase** | 0.816 | |  | | **0.824** |  | 0.350 |
| **Cdc19p, Pyruvate kinase** | **0.254** | |  | | **0.398** |  | **0.127** |
| **Dak1p, Dihydroxyacetone kinase** | **0.328** | |  | | **0.559** |  | 0.226 |
| **Eno1p, Enolase** | **0.550** | |  | | 0.794 |  | **0.202** |
| **Eno2p, Enolase II** | **0.372** | |  | | **0.592** |  | 0.347 |
| **Fba1p, Fructose 1, 6-bisphosphate aldolase** | **0.330** | |  | | **0.543** |  | **0.305** |
| Fbp1p, Fructose-1, 6-bisphosphatase |  | |  | |  |  | 0.218 |
| Fum1p, Fumarase |  | |  | |  |  | 0.413 |
| Gcy1p, NADP(+) coupled glycerol dehydrogenase | 0.331 | |  | | 0.589 |  | 0.238 |
| **Glc3p, Glycogen branching enzyme** | **0.383** | |  | | **0.508** |  | **0.178** |
| **Glk1p, Glucokinase** | **0.317** | |  | | **0.538** |  | **0.244** |
| **Gnd1p, 6-phosphogluconate dehydrogenase** | **0.247** | |  | | **0.407** |  | **0.272** |
| **Gor1p, Glyoxylate reductase** |  | |  | |  |  | **0.205** |
| **Gpd1p, Glycerol-3-phosphate dehydrogenase** | | |  | |  |  | **0.164** |
| **Gph1p, Glycogen phosphorylase** | **0.281** | |  | | **0.349** |  |  |
| **Gpm1p, Tetrameric phosphoglycerate mutase** | **0.268** | |  | | **0.451** |  | **0.143** |
| **Hxk1p, Hexokinase isoenzyme** | **0.311** | |  | | **0.523** |  | 0.584 |
| **Hxk2p, Hexokinase isoenzyme** | **0.327** | |  | | **0.478** |  | **0.334** |
| **Icl1p, Isocitrate lyase** | **0.539** | |  | | **0.790** |  | **0.192** |
| **Idi1p, Isopentenyl diphosphate** | 0.420 | |  | | 0.790 |  | **0.391** |
| **Idp2p, NADP-specific isocitrate dehydrogenase** | **0.604** | |  | | 1.028 |  | **0.193** |
| **Mdh1p, Mitochondrial malate dehydrogenase** | **0.492** | |  | | 0.739 |  | 0.453 |
| **Mdh2p, Malate dehydrogenase** | **0.259** | |  | | **0.410** |  | **0.095** |
| **Mls1p, Malate synthase** | 0.387 | |  | | 0.592 |  | **0.265** |
| **Pck1p, Phosphoenolpyruvate carboxykinase** | 0.503 | |  | | 0.677 |  | **0.144** |
| **Pdc1p, Major pyruvate decarboxylase** | **0.225** | |  | | **0.354** |  | **0.172** |
| Pfk1p, Phosphofructokinase | 0.357 | |  | | 0.531 |  | 0.307 |
| **Pfk2p, 6-Phosphofructo-2-kinase** | **0.372** | |  | | **0.553** |  | 0.254 |
| **Pgi1p, Phosphoglucose isomerase** | **0.363** | |  | | **0.597** |  | **0.214** |
| **Pgk1p, 3-Phosphoglycerate kinase** | **0.285** | |  | | **0.490** |  | **0.213** |
| **Pgm2p, Phosphoglucomutase** | **0.500** | |  | | **0.799** |  | **0.268** |
| **Pmi40p, Mannose-6-phosphate isomerase** | **0.258** | |  | | **0.508** |  | **0.370** |
| **Psa1p, GDP-mannose pyrophosphorylase** | **0.234** | |  | | **0.399** |  | **0.167** |
| **Pyc2p, Pyruvate carboxylase** | **0.369** | |  | | **0.572** |  | 0.150 |
| **Rhr2p, Isoform of DL-glycerol-3-phosphatase** | **0.355** | |  | | **0.582** |  | **0.411** |
| Sol3p, 6-Phosphogluconolactonase |  | |  | |  |  | 0.497 |
| **Sol4p, 6-Phosphogluconolactonase** | **0.640** | |  | | 0.807 |  | **0.300** |
| **Suc2p, Invertase** | **0.605** | |  | | 1.030 |  | **0.254** |
| **Tal1p, Transaldolase** | **0.214** | |  | | **0.346** |  | **0.151** |
| **Tdh1p, Glyceraldehyde-3-phosphate dehydrogenase** | **0.324** | |  | | **0.501** |  | **0.138** |
| **Tdh2p, Glyceraldehyde-3-phosphate dehydrogenase** | 0.413 | |  | | **0.532** |  | 0.249 |
| **Tdh3p, Glyceraldehyde-3-phosphate dehydrogenase** | **0.202** | |  | | 0.392 |  | **0.172** |
| **Tkl1p, Transketolase** | **0.338** | |  | | **0.426** |  | **0.248** |
| **Tkl2p, Transketolase** | 0.670 | |  | | 0.960 |  | **0.137** |
| **Tpi1p, Triose phosphate isomerase** | **0.505** | |  | | 0.870 |  | **0.354** |
| **Ypr1p, Yeast putative reductase** | **0.500** | |  | | **0.750** |  | **0.351** |
| **Zwf1p, Glucose-6-phosphate dehydrogenase** | **0.254** | |  | | **0.445** |  | **0.172** |
|  |  | |  | |  |  |  |
| **B. Amino Acid Metabolism** |  | |  | |  |  |  |
| **Aap1p, Arginine/alanine aminopeptidase** |  | |  | |  |  | **0.329** |
| Aat2p, Aspartate aminotransferase | 0.296 | |  | | 0.409 |  | 0.169 |
| **Ala1p, Alanyl-tRNA synthetase** | **0.293** | |  | | **0.510** |  | **0.224** |
| **Arg1p, Arginosuccinate synthetase** | **0.195** | |  | | **0.336** |  |  |
| Aro2p, Chorismate synthase and flavin reductase | 0.197 | |  | | 0.317 |  |  |
| **Aro4p, 3-Deoxy-D-arabino-heptulosonate-7-phosphate synthase** | **0.259** | |  | | **0.444** |  | 0.215 |
| Aro7p, Chorismate mutase |  | |  | |  |  | 0.269 |
| **Aro8p, Aromatic aminotransferase I** | **0.347** | |  | | **0.596** |  | **0.311** |
| **Aro9p, Aromatic aminotransferase II** | 0.411 | |  | | 0.699 |  | **0.314** |
| **Asn1p, Asparagine synthetase** | **0.306** | |  | | **0.449** |  | **0.249** |
| **Asn2p, Asparagine synthetase** | **0.281** | |  | | **0.395** |  | **0.202** |
| **Car1p, Arginase** | 0.365 | |  | | 0.622 |  | **0.264** |
| **Car2p, Catabolism of arginine** | **0.338** | |  | | **0.619** |  | **0.213** |
| **Cdc60p, Leucyl tRNA synthetase** | **0.291** | |  | | **0.487** |  | **0.235** |
| **Cpa2p, Carbamyl phosphate synthetase A** | **0.368** | |  | | **0.514** |  | 0.406 |
| **Cys3p, Cystathionine gamma-lyase** | **0.267** | |  | | **0.407** |  | **0.246** |
| **Cys4p, Cystathionine beta-synthase** | **0.264** | |  | | **0.434** |  | **0.180** |
| **Ded81p, Asparaginyl-tRNA synthetase** | 0.236 | |  | | 0.427 |  | **0.182** |
| **Dps1p, Aspartyl-tRNA synthetase** | **0.403** | |  | | 0.616 |  | **0.110** |
| **Dug1p, Deficient in utilization of glutathione** | **0.327** | |  | | **0.558** |  | **0.212** |
| Fsh1p, Serine hydrolases | 0.453 | |  | | 0.675 |  | **0.266** |
| **Gas1p, Beta-1, 3-glucanosyltransferase** | **0.369** | |  | | **0.511** |  | 0.307 |
| **Gdh1p, Glutamate dehydrogenase** | **0.326** | |  | | **0.473** |  | **0.185** |
| Gln1p, Glutamine synthetase | 0.504 | |  | | 0.833 |  |  |
| Gln4p, Glutamine tRNA synthetase |  | |  | |  |  | 0.268 |
| **Grs1p, Glycyl-tRNA synthase** | 0.229 | |  | | **0.470** |  | **0.181** |
| **Gus1p, Glutamyl-tRNA synthetase** | 0.675 | |  | | 0.646 |  | **0.165** |
| His4p, Phosphoribosyl-ATP pyrophosphatase and histidinol dehydrogenase | 0.366 | |  | | 0.767 |  | 0.212 |
| His7p, Imidazole glycerol phosphate synthase |  | |  | |  |  | 0.205 |
| **Hom2p, Aspartic beta semi-aldehyde dehydrogenase** | **0.251** | |  | | **0.405** |  | **0.201** |
| **Hom6p, Homoserine dehydrogenase** | **0.269** | |  | | **0.466** |  | **0.192** |
| **Ils1p, Isoleucine-tRNA synthetase** | **0.298** | |  | | **0.460** |  | **0.314** |
| Krs1p, Lysine--tRNA ligase |  | |  | |  |  | 0.183 |
| **Lap2p, Leucine aminopeptidases** | **0.435** | |  | | **0.512** |  | 0.231 |
| Leu1p, Isopropylmalate isomerase | 0.341 | |  | | 0.257 |  | 0.459 |
| **Leu4p, Alpha-isopropylmalate synthase** | **0.324** | |  | | 0.491 |  |  |
| **Met6p, Cobalamin-independent methionine synthase** | **0.437** | |  | | **0.625** |  | **0.241** |
| **Met17p, Methionine and cysteine synthase** | **0.325** | |  | | **0.451** |  | **0.179** |
| **Mmf1p, Mitochondrial matrix factor** |  | |  | |  |  | **0.427** |
| Pro2p, Gamma-glutamyl phosphate reductase | 0.435 | |  | | 0.591 |  | 0.203 |
| **Sah1p, S-adenosyl-L-homocysteine hydrolase** | **0.252** | |  | | **0.354** |  | **0.273** |
| **Sam1p, S-adenosylmethionine synthetase** | **0.291** | |  | | **0.455** |  | **0.268** |
| **Sam4p, S-adenosylmethionine-homocysteine methyltransferase** | | | | | |  | **0.333** |
| Ser1p, 3-Phosphoserine aminotransferase | 0.371 | |  | | 0.465 |  | 0.170 |
| **Ses1p, Seryl-tRNA synthetase** | 0.212 | |  | | 0.419 |  | **0.190** |
| **Spe3p, Spermidine synthase** | **0.322** | |  | | **0.671** |  | **0.356** |
| **Thr1p, Homoserine kinase** | **0.344** | |  | | **0.500** |  | 0.210 |
| **Thr4p, Threonine synthase** | **0.304** | |  | | **0.534** |  | **0.334** |
| Trp2p, Anthranilate synthase | 0.248 | |  | | 0.407 |  | 0.259 |
| **Trp5p, Tryptophan synthase** | 0.282 | |  | | 0.468 |  | **0.208** |
| Uga1p, Gamma-aminobutyrate (GABA) transaminase | | |  | |  |  | 0.213 |
| **Vas1p, Valyl-tRNA synthetase** | **0.351** | |  | | **0.530** |  | **0.244** |
|  |  | |  | |  |  |  |
| **C. Nucleotide Metabolism** |  | |  | |  |  |  |
| **Ade1p, N-succinyl-5-aminoimidazole-4-carboxamide ribotide synthetase** | 0.691 | |  | | 0.867 |  | **0.405** |
| **Ade5, 7p, Aminoimidazole ribotide/glycinamide ribotide synthase** | | | | | |  | **0.265** |
| **Ade6p, Formylglycinamidine-ribonucleotide-synthetase** | | | | |  |  | **0.251** |
| Ade12p, Adenylosuccinate synthase |  | |  | |  |  | 0.236 |
| Ade13p, Adenylosuccinate lyase | 0.269 | |  | | 0.348 |  | 0.277 |
| **Ade16p, 5-Aminoimidazole-4-carboxamide ribonucleotide transformylase** | **0.254** | |  | | **0.355** |  |  |
| Ade17p, 5-Aminoimidazole-4-carboxamide ribonucleotide transformylase | | | | | |  | 0.490 |
| **Adk1p, Adenylate kinase** | **0.369** | |  | | **0.658** |  | **0.324** |
| **Ado1p, Adenosine kinase** | **0.424** | |  | | **0.676** |  | **0.407** |
| **Apa1p, Diadenosine 5',5''-P1,P4-tetraphosphate phosphorylase** | 0.269 | |  | | 0.403 |  | **0.177** |
| **Apt1p, Adenine phosphoribosyltransferase** |  | | | |  |  | **0.282** |
| **Bna1p, 3-Hydroxyanthranilic acid dioxygenase** | **0.343** | |  | | 0.671 |  | 0.232 |
| Fcy1p, Deaminase | 0.339 | |  | | 0.690 |  | 0.319 |
| **Gua1p, GMP synthase** | 0.256 | |  | | 0.415 |  | **0.259** |
| **Guk1p, Guanylate kinase** | **0.305** | |  | | **0.550** |  | **0.244** |
| **Ham1p, Deoxyribonucleoside triphosphate pyrophosphohydrolase** | | | | | |  | **0.364** |
| Hnt1p, Adenosine 5'-monophosphoramidase | 0.398 | |  | | 0.672 |  | 0.467 |
| **Hpt1p, Hypoxanthine guanine phosphoribosyltransferase** | | | | |  |  | **0.268** |
| **Rnr2p, Ribonucleotide reductase** | **0.362** | |  | | **0.567** |  | **0.197** |
| **Rnr4p, Ribonucleotide-diphosphate reductase** | **0.362** | |  | | **0.441** |  | **0.183** |
| **Ura1p, Dihydroorotate dehydrogenase** | **0.534** | |  | | 0.977 |  | **0.309** |
| **Ura2p, Bifunctional carbamoylphosphate synthetase** | **0.509** | |  | | 0.603 |  | **0.221** |
| **Ura4p, Dihydroorotase** |  | |  | |  |  | **0.315** |
| **Ura5p, Major orotate phosphoribosyltransferase** | 0.589 | |  | | 0.778 |  | **0.301** |
| **Ura6p, Uridylate kinase** | 0.248 | |  | | 0.481 |  | **0.331** |
| **Ynk1p, Yeast nucleoside diphosphate kinase** | **0.355** | |  | | **0.562** |  | **0.198** |
|  |  | |  | |  |  |  |
| **D. Lipid Metabolism** |  | |  | |  |  |  |
| **Acb1p, Acyl-CoA-binding** | **0.513** | |  | | 0.646 |  | **0.615** |
| **Erg10p, Acetyl-CoA C-acetyltransferase** | 0.321 | |  | | **0.404** |  | 0.140 |
| **Erg13p, HMG-CoA synthase** | **0.200** | |  | | **0.363** |  | **0.167** |
| **Erg20p, Farnesyl pyrophosphate synthetase** | 0.542 | |  | | 0.575 |  | **0.340** |
| **Kes1p, Oxysterol binding protein family** | **0.276** | |  | | **0.392** |  | 0.141 |
| Mvd1p, Mevalonate pyrophosphate decarboxylase | 0.320 | |  | | 0.498 |  | 0.305 |
| **Pdx3p, Pyridoxine (pyridoxamine) phosphate oxidase** | **0.312** | |  | | **0.533** |  | **0.244** |
| **Sec14p, Phosphatidylinositol/phosphatidylcholine transfer protein** | **0.360** | |  | | **0.532** |  | **0.249** |
|  |  | |  | |  |  |  |
| **E. Oxidative Stress** |  | |  | |  |  |  |
| **Ahp1p, Thiol-specific peroxiredoxin** | **0.387** | |  | | **0.665** |  | **0.459** |
| **Ccs1p, Copper chaperone for Sod1p** | 0.421 | |  | | 0.560 |  | **0.261** |
| Glr1p, Glutathione oxidoreductase | 0.379 | |  | | 0.571 |  | 0.172 |
| **Gre3p, Aldose reductase** | 0.486 | |  | | 0.707 |  | **0.234** |
| Grx1p, Hydroperoxide | 0.539 | |  | | 0.741 |  | 0.272 |
| Grx2p, Glutaredoxin; thioltransferase | 0.390 | |  | | 0.715 |  | 0.602 |
| Grx3p, Superoxide-radical responsive oxidoreductas |  | | | |  |  | 0.263 |
| **Hyr1p, Thiol peroxidase** | **0.376** |  | | 0.645 | |  | **0.157** |
| Mcr1p, Mitochondrial NADH-cytochrome b5 reductase |  | | | |  |  | 0.200 |
| Mxr1p, Methionine-S-sulfoxide reductase | 0.337 | |  | | 0.803 |  | 0.478 |
| **Oye2p, NADPH oxidoreductase** | **0.335** | |  | | **0.456** |  | 0.300 |
| Prx1p, Peroxiredoxin | 0.366 | |  | | 0.645 |  | 0.258 |
| **Sod1p, Superoxide dismutase** | **0.394** | |  | | **0.740** |  | **0.477** |
| **Sod2p, Mitochondrial superoxide dismutase** | 0.503 | |  | | 0.721 |  | **0.467** |
| **Svf1p, Protein with a role in cell survival pathways** | **0.380** | |  | | **0.413** |  | 0.223 |
| **Trr1p, Thioredoxin reductase-like protein** | **0.351** | |  | | **0.446** |  | 0.205 |
| **Trx1p, Thioredoxin isoenzyme** | 0.466 | |  | | 0.790 |  | **0.476** |
| Trx2p, Thioredoxin isoenzyme | 0.440 | |  | | 0.912 |  | 0.548 |
| **Tsa1p, Thiol-specific antioxidant** | **0.242** | |  | | **0.344** |  | **0.155** |
| Yhb1p, Nitric oxide oxidoreductase | 0.460 | |  | | 0.615 |  | 0.516 |
|  |  | |  | |  |  |  |
| **F. Protein Folding** |  | |  | |  |  |  |
| Aha1p, Activator of heat shock protein 90 ATPase |  | | | |  |  | 0.307 |
| Cdc37p, Essential Hsp90p co-chaperone | 0.162 | |  | | 0.272 |  | 0.335 |
| **Cpr1p, Peptidyl-prolyl cis-trans isomerase (cyclophilin)** | **0.413** | |  | | 0.904 |  | **0.443** |
| **Cpr3p, Cyclosporin-sensitive proline rotamase** |  | | | |  |  | **0.182** |
| Cpr5p, Cyclosporin-sensitive proline rotamase |  | | | |  |  | 0.326 |
| **Cpr6p, Peptidyl-prolyl cis-trans isomerase** | 0.396 | |  | | 0.448 |  | **0.234** |
| **Fpr1p, Peptidyl-prolyl cis-trans isomerase** | **0.461** | |  | | **0.814** |  | **0.634** |
| Hch1p, Heat shock protein regulator |  | |  | |  |  | 0.414 |
| **Hsc82p, Chaperone of the Hsp90 family** | **0.204** | |  | | **0.429** |  | **0.248** |
| **Hsp12p, Heat shock protein** | **0.674** | |  | | **1.487** |  | **0.416** |
| **Hsp26p, Heat shock protein** | **0.374** | |  | | **0.563** |  | **0.124** |
| Hsp31p, Heat shock protein | 0.524 | |  | | 1.189 |  | 0.422 |
| Hsp42p, Heat shock protein |  | |  | |  |  | 0.100 |
| **Hsp82p, Heat shock protein** | **0.213** | |  | | **0.294** |  | 0.117 |
| **Hsp104, Heat shock protein** | **0.317** | |  | | **0.448** |  | **0.109** |
| **Ssa1p, Hsp70 family** | 0.468 | |  | | **0.589** |  | **0.135** |
| Ssa2p, Hsp70 family | 0.310 | |  | | 0.465 |  | 0.010 |
| **Ssa3p, Hsp70 family** |  | |  | |  |  | **0.169** |
| **Ssa4p, Hsp70 family** | **0.596** | |  | | **0.774** |  | 0.201 |
| **Sba1p, Co-chaperone** | **0.386** | |  | | 0.700 |  | **0.369** |
| **Ssb2p, Hsp70 family** | **0.249** | |  | | **0.374** |  | 0.235 |
| **Ssc1p, Heat shock protein** | **0.361** | |  | | **0.558** |  | **0.225** |
| **Sse1p, Member of the HSP70 family** | **0.287** | |  | | **0.417** |  | **0.159** |
| Sse2p, Member of the HSP70 family |  | |  | |  |  | 0.367 |
| **Sti1p, Hsp90 co-chaperone** | **0.347** | |  | | **0.550** |  | **0.195** |
|  |  | |  | |  |  |  |
| **G. Other Functions** |  | |  | |  |  |  |
| **Abp1p, Actin-binding protein** | **0.304** | |  | | **0.471** |  | **0.146** |
| Abp140p, Actin binding protein |  | |  | |  |  | 0.268 |
| Ach1p, Protein with CoA transferase activity | 0.402 | |  | | 0.670 |  | 0.416 |
| **Acs1p, Acetyl-coA synthetase** | **0.456** | |  | | **0.710** |  | **0.141** |
| **Act1p, Actin** | **0.257** | |  | | **0.370** |  | **0.208** |
| **Ape2p, Aminopeptidase yscII** | **0.358** | |  | | **0.514** |  | **0.238** |
| **Arf1p, ADP-ribosylation factor 1** | **0.470** | |  | | 0.748 |  | 0.259 |
| Arf2p, ADO-ribosylation factor 2 |  | |  | |  |  | 0.130 |
| **Asc1p, Guanine nucleotide dissociation inhibitor for Gpa2p** | **0.328** | |  | | **0.512** |  | **0.151** |
| **Bat2p, Branched-chain amino acid aminotransferase** | | |  | |  |  | **0.193** |
| **Bdh1p, NAD-dependent (R,R)-butanediol dehydrogenase** | **0.217** | |  | | **0.372** |  |  |
| **Bgl2p, Beta-glucanase** | **0.612** | |  | | 0.908 |  | **0.276** |
| Bmh1p, 14-3-3 protein | 0.514 | |  | | 0.589 |  | 0.312 |
| Bmh2p, 14-3-3 protein, minor isoform | 0.378 | |  | | 0.608 |  | 0.238 |
| **Cdc33p, mRNA cap binding protein/translation factor eIF4E** | **0.275** | |  | | **0.435** |  | **0.200** |
| **Cdc48p, ATPase** | **0.331** | |  | | **0.360** |  | 0.110 |
| Cmd1p, Calmodulin |  | |  | |  |  | 0.287 |
| **Cof1p, Cofilin** | **0.292** | |  | | **0.410** |  | **0.186** |
| **Dcs1p, Hydrolase involved in mRNA decapping** | | |  | |  |  | 0.195 |
| Dcs2p, Decapping scavenger | 0.326 | |  | | 0.628 |  | 0.890 |
| **Ddr48p, DNA damage-responsive protein** | **0.245** | |  | | **0.434** |  |  |
| Dsf1p, Deletion suppressor of mpt5 mutation |  | |  | |  |  | 0.267 |
| **Eft2p, Elongation factor 2 (EF-2)** | **0.329** | |  | | **0.466** |  | **0.160** |
| Egd2p, Subunit of the polypeptide-associated complex | 0.232 | |  | | 0.513 |  | 0.115 |
| **Emi2p, Regulator for IME1 transcription factor** | **0.217** | |  | | **0.413** |  | 0.160 |
| **Exg1p, Exo-1, 3-beta-glucanase** | 0.768 | |  | | 0.951 |  | **0.358** |
| **Gdi1p, GDP dissociation inhibitor** | **0.277** | |  | | **0.458** |  | 0.176 |
| Get3p, Guanine nucleotide exchange factor for Gpa1p | | |  | |  |  | 0.207 |
| **Glc7p, Type 1 serine/threonine protein phosphatase** | 0.379 | |  | | **0.359** |  |  |
| **Glo1p, Glyoxalase** | **0.398** | |  | | **0.601** |  | 0.222 |
| Gre1p, Hydrophilin | 0.231 | |  | | 0.664 |  | 0.069 |
| **Gsp2, GTP binding protein** | **0.448** | |  | | **0.617** |  | **0.321** |
| Hem12p, Heme biosynthesis proteoin |  | |  | |  |  | 0.188 |
| **Hmf1p, Homologous Mmf1p factor** |  | |  | |  |  | **0.372** |
| Hor2p, Glycerol-1-phosphatase |  | |  | |  |  | 0.588 |
| **Hyp2p, Translation elongation factor** | **0.251** | |  | | **0.452** |  | 0.235 |
| **Ipp1p, Inorganic pyrophosphatase (PPase),** | **0.388** | |  | | **0.675** |  | **0.280** |
| Kap123, Karyopherin beta |  | |  | |  |  | 0.211 |
| Lia1p, Deoxyhypusine hydroxylase |  | |  | |  |  | 0.260 |
| Mlc1, Light chain for Myo1p | 0.397 | |  | | 0.514 |  | 0.458 |
| **Mrp8p, Mitochondrial ribosomal protein** | 0.645 | |  | | 0.663 |  | **0.151** |
| Npc2p, Functional homolog of human NPC2/He1 | 0.267 | |  | | 0.271 |  | 0.559 |
| Npt1p, Nicotinate phosphoribosyl transferase | 0.351 | |  | | 0.550 |  | 0.346 |
| **Nqm1p, Transaldolase of unknown function** |  | | | |  |  | **0.134** |
| Ntf2p, Nuclear envelope protein |  | |  | |  |  | 0.407 |
| Paa1p, Polyamine acetyltransferase |  | |  | |  |  | 0.241 |
| **Pab1p, Poly(A) binding protein** | **0.275** | |  | | **0.361** |  | **0.185** |
| **Pai3p, Proteinase A inhibitor** | **0.645** | |  | | 1.636 |  | 0.451 |
| **Pbi2p, Proteinase B inhibitor** | **0.691** | |  | | **1.554** |  | **0.475** |
| **Pdx3p, Pyridoxine (pyridoxamine) phosphate oxidase** | **0.312** | |  | | **0.533** |  |  |
| **Pep4, Vacuolar aspartyl protease** | 0.397 | |  | | **0.616** |  | 0.304 |
| Pfy1p, Profilin | 0.361 | |  | | 0.644 |  | 0.170 |
| **Pho3p, Constitutively expressed acid phosphatase** | **0.680** | |  | | 0.985 |  | 0.351 |
| Pho5p, Repressible acid phosphatase |  | |  | |  |  | 0.289 |
| **Pho12p, Repressible acid phosphatases** | 0.782 | |  | | 1.111 |  | **0.377** |
| **Pil1p, Component of eisosomes** | 0.274 | |  | | **0.352** |  |  |
| Pir3p, O-glycosylated covalently-bound cell wall protein |  | | | |  |  | 0.304 |
| **Pnc1p, Nicotinamidase** | **0.393** | |  | | 0.704 |  | **0.257** |
| **Pol30p, Proliferating cell nuclear antigen (PCNA)** | 0.315 | |  | | 0.476 |  | **0.315** |
| Por1p, Mitochondrial porin | 0.443 | |  | | 0.662 |  |  |
| **Prb1p, Vacuolar proteinase B** | **0.387** | |  | | **0.644** |  | **0.247** |
| Prc1p, Vacuolar proteinase C |  | |  | |  |  | 0.010 |
| **Prd1p, Zinc metalloendopeptidase** |  | |  | |  |  | **0.312** |
| **Pst2p, Protein with similarity to flavodoxin-like proteins** | **0.393** | |  | | 0.629 |  | **0.242** |
| **Pub1p, Poly (A)+ RNA-binding protein** |  | |  | |  |  | **0.233** |
| Ras2p, GTP-binding protein | 0.370 | |  | | 0.774 |  | 0.442 |
| Rdi1p, Rho GDP dissociation inhibitor | 0.388 | |  | | 0.531 |  | 0.198 |
| **Rdl1p, Rhodanese-Like protein** | **0.410** | |  | | 0.815 |  | **0.310** |
| **Ree1p, Regulation of enolase** | **0.628** | |  | | 1.039 |  | **0.462** |
| Rfs1p, Rad55 (Fifty-five) suppressor |  | |  | |  |  | 0.256 |
| Rgi1p, Respiratory Growth Induced protein | 0.362 | |  | | 0.712 |  | 0.577 |
| Rib3p, 3,4-Dihydroxy-2-butanone-4-phosphate synthase | 0.272 | |  | | 0.525 |  | 0.392 |
| Rib4p, Lumazine synthase | 0.480 | |  | | 0.522 |  | 0.121 |
| Rna1p, Ran GTPase activating protein |  | |  | |  |  | 0.010 |
| **Rpp0p, Ribosomal protein** | **0.380** | |  | | 0.624 |  | 0.195 |
| **Rps0bp, Ribosomal protein** | **0.308** | |  | | **0.420** |  | 0.114 |
| Rps1ap, Ribosomal protein |  | |  | |  |  | 0.104 |
| Rps1bp, Ribosomal protein | 0.309 | |  | | 0.557 |  |  |
| **Rps3p, Ribosomal protein** | 0.312 | |  | | **0.546** |  |  |
| Rps5p, Ribosomal protein | 0.497 | |  | | 0.606 |  | 0.160 |
| **Rps12p, Ribosomal protein** | **0.207** | |  | | **0.347** |  |  |
| Rps20p, Ribosomal protein | 0.212 | |  | | 0.330 |  |  |
| **Rps21ap, Ribosomal protein** | 0.301 | |  | | 0.517 |  | **0.169** |
| Rps21bp, Ribosomal protein | 0.260 | |  | | 0.357 |  |  |
| **Rps31p, Ribosomal protein** | **0.404** | |  | | **0.649** |  | **0.414** |
| **Rpl5p, Ribosomal protein** | 0.636 | |  | | 0.638 |  | **0.192** |
| **Rpl10p, Ribosomal protein** | **0.347** | |  | | **0.580** |  |  |
| **Rpl20ap, Ribosomal protein** | **0.327** | |  | | 0.505 |  |  |
| Rpl22ap, Ribosomal protein |  | |  | |  |  |  |
| Rpl25p, Ribosomal protein | 0.559 | |  | | 0.718 |  | 0.213 |
| **Rpl27bp, Ribosomal protein** | 0.322 | |  | | 0.713 |  | **0.094** |
| **Rpl36bp, Ribosomal protein** | **0.402** | |  | | 0.646 |  |  |
| **Rtc3p, Restriction of telomere capping** | **0.456** | |  | | 0.901 |  | **0.477** |
| Sac6p, Fimbrin, actin-bundling protein |  | |  | |  |  | 0.170 |
| **Sar1p, GTPase** | 0.370 | |  | | 0.532 |  | 0.193 |
| **Scw4p, Cell wall protein with similarity to glucanases** | 0.600 | |  | | 1.094 |  | **0.222** |
| Sec53p, Phosphomannomutase |  | |  | |  |  | 0.411 |
| **Sfa1p, Alcohol and formaldehyde dehydrogenase** | 0.391 | |  | | 0.618 |  | **0.248** |
| **Sgt2p, Glutamine-rich cytoplasmic protein** | **0.306** | |  | | **0.485** |  | **0.184** |
| Shp1p, Suppressor of high-copy PP1 |  | |  | |  |  | 0.160 |
| **Skp1p, Suppressor of kinetochore protein mutant** |  | | | |  |  | **0.224** |
| Sip18p, Salt induced protein |  | |  | |  |  | 0.125 |
| Smt3p, Ubiquitin-like protein of the SUMO family | 0.458 | |  | | 0.773 |  | 0.490 |
| Spg4p, Stationary phase gene |  | |  | |  |  | 0.113 |
| Sub2p, Component of TREX required for mRNA export | 0.353 | |  | | 0.497 |  |  |
| Sui1p, Translation initiation factor eIF1 | 0.362 | |  | | 0.502 |  | 0.486 |
| **Tef2p, Translational elongation factor EF-1 alpha** | 0.438 | |  | | **0.505** |  | **0.126** |
| **Tfs1p, Protein that inhibits carboxypeptidase Y and Ira2p** | 0.432 | |  | | 0.917 |  | **0.369** |
| **Tif2p, Translation initiation factor eIF4A** | **0.419** | |  | | **0.535** |  | **0.275** |
| Tif3p, Translation initiation factor eIF-4B | 0.209 | |  | | 0.378 |  | 0.281 |
| Tif6p, Translation initiation factor 6 (eIF6) |  | |  | |  |  | 0.195 |
| **Tma19p, Protein that associates with ribosomes** | **0.274** | |  | | **0.503** |  | **0.291** |
| **Tmt1p, Trans-aconitate methyltransferase** | **0.499** | |  | | 0.980 |  | 0.288 |
| **Tpm1p, Major isoform of tropomyosin** | **0.321** | |  | | **0.561** |  | **0.323** |
| Tub1p, Alpha-tubulin | 0.487 | |  | | 0.633 |  | 0.130 |
| **Tub2p, Tubulin beta chain** | **0.461** | |  | | **0.499** |  | **0.164** |
| **Uba1p, Ubiquitin activating enzyme** | **0.304** | |  | | **0.461** |  | **0.247** |
| Ubc4p, Ubiquitin-conjugating enzyme |  | |  | |  |  | 0.357 |
| Uth1p, Protein involved in cell wall biogenesis |  | |  | |  |  | 0.214 |
| **Vma1p, Subunit of vacuolar H+-ATPase** | **0.348** | |  | | **0.468** |  | **0.181** |
| **Vma2p, Subunit of vacuolar H+-ATPase** | **0.307** | |  | | **0.415** |  |  |
| **Vma5p, Subunit of vacuolar H+-ATPase** | 0.390 | |  | | **0.404** |  | 0.056 |
| **Wtm1p, Transcriptional modulator** | **0.336** | |  | | **0.470** |  | **0.265** |
| Wwm1p, Protein interacting with metacaspase | 0.518 | |  | | 0.436 |  | 0.118 |
| **Ycp4p, Protein of unknown function** |  | |  | |  |  | **0.362** |
| Ygp1p, Cell wall-related secretory glycoprotein |  | | | |  |  | 0.320 |
| **Ykt6p, Vesicle protein** | 0.280 | |  | | 0.470 |  | **0.295** |
| Ypd1p, Phosphorelay intermediate protein |  | |  | |  |  | 0.355 |
| **Yrb1p, Yeast Ran binder** | **0.243** | |  | | **0.372** |  | **0.137** |
| **Ysa1p, Nudix hydrolase family member** |  | |  | |  |  | **0.330** |
| **Zeo1p, Peripheral protein of the plasma membrane** | **0.452** | |  | | 0.859 |  | 0.492 |
| **Zpr1p, Zinc finger protein** | 0.446 | |  | | 0.648 |  | **0.252** |
| **YBL036C, Hypothetical protein** |  | |  | |  |  | **0.365** |
| YBR053C, Hypothetical protein |  | |  | |  |  | 0.374 |
| **YBR056W, Hypothetical protein** |  | |  | |  |  | **0.241** |
| YBR085C-A, Hypothetical protein |  | |  | |  |  | 0.356 |
| **YEL047C, Hypothetical protein** |  | |  | |  |  | 0.374 |
| **YDR341C, Hypothetical protein** | **0.314** | |  | | **0.528** |  | **0.266** |
| YDL086W, Hypothetical protein |  | |  | |  |  | 0.269 |
| YDL124W, Hypothetical protein | 0.380 | |  | | 0.560 |  | 0.341 |
| YHR138C, Hypothetical protein |  | |  | |  |  | 0.413 |
| YJL055W, Hypothetical protein |  | |  | |  |  | 0.470 |
| **YJL068C, Hypothetical protein** | 0.443 | |  | | 0.942 |  | **0.275** |
| YKR043C, Hypothetical protein |  | |  | |  |  | 0.174 |
| **YKR043C, Hypothetical protein** | **0.314** | |  | | **0.545** |  | 0.272 |
| YLR301W, Hypothetical protein | 0.555 | |  | | 0.525 |  | 0.286 |
| YML079W, Hypothetical protein |  | |  | |  |  | 0.475 |
| **YMR027W, Hypothetical protein** |  | |  | |  |  | **0.282** |
| YMR090W, Hypothetical protein |  | |  | |  |  | 0.280 |
| **YMR099C, Hypothetical protein** | 0.376 | |  | | 0.613 |  | **0.345** |
| **YNR034W-A, Hypothetical protein** | **0.463** | |  | | 0.798 |  | **0.418** |
| **YMR226C, Hypothetical protein** | **0.330** | |  | | **0.511** |  | 0.233 |
| YNL010W, Hypothetical protein | 0.304 | |  | | 0.575 |  | 0.199 |
| **YNL134C, Hypothetical protein** | 0.536 | |  | | **0.669** |  | **0.432** |
| YOL057W, Hypothetical protein |  | |  | |  |  | 0.489 |
| YOR021C, Hypothetical protein | 0.636 | |  | | 0.745 |  | 0.370 |
| **YPL225W, Hypothetical protein** |  | |  | |  |  | **0.239** |
| **YPR127W, Hypothetical protein** | **0.385** | |  | | 0.730 |  | **0.268** |
| YPR127W, Hypothetical protein |  | |  | |  |  | 0.123 |

Proteins were classified into carbohydrate metabolism (A), amino acid metabolism (B), nucleotide metabolism (C), lipid metabolism (D), oxidative stress (E), protein folding (F), and proteins with other functions (G). In iTRAQ1, we used 115/113 (t30_a_/t0_a_) and 116/114 (t30_b_/30_b_) ratios to examine proteins that changed levels in response to glucose re-feeding. In iTRAQ2, we used 114/113 (t30_c_/t0_c_) to identify proteins that changed levels following glucose addition. Individual t30/t0 ratio that had p-values less than 0.05 were highlighted in bold. Proteins that had one of the three t30/t0 ratios with p-values smaller than 0.05 were highlighted in bold.
